# Nurses’ Engagement in Antimicrobial Stewardship Programmes: A Mapping Review of Influencing Factors Based on Irvine’s Theory

**DOI:** 10.3390/nursrep15060216

**Published:** 2025-06-12

**Authors:** Susana Filipe, Paulo Santos-Costa, Celeste Bastos, Amélia Castilho

**Affiliations:** 1 Health Sciences Research Unit: Nursing (UICISA: E), Nursing School of Coimbra (ESEnfC), 3046-851 Coimbra, Portugal; paulocosta@esenfc.pt (P.S.-C.); afilomena@esenfc.pt (A.C.); 2 Local Health Unit of Baixo Mondego, 3094-001 Figueira da Foz, Portugal; 3 CINTESIS@RISE, Nursing School of Porto (ESEP), 4200-072 Porto, Portugal; cbastos@esenf.pt

**Keywords:** antimicrobial, antimicrobial resistance, antimicrobial stewardship, barriers, facilitators, nursing, nursing role effectiveness model, nursing-sensitive outcomes

## Abstract

Antimicrobial resistance (AMR) is a pressing global health challenge, driving the need for effective antimicrobial stewardship (AMS) programmes. Despite nurses’ critical role in care delivery, their involvement in AMS remains under-recognized. **Objectives**: This mapping review aims to identify barriers and facilitators influencing nurses’ engagement in AMS programmes and examine nursing-sensitive outcomes associated with their participation, using Irvine’s Nursing Role Effectiveness Model (NREM) as a guiding framework. **Methods**: A systematic mapping review was conducted following Joanna Briggs Institute (JBI) guidance and reported using the PRISMA-ScR checklist. The protocol was registered on the Open Science Framework. Searches were conducted in MEDLINE, CI-NAHL, Scopus, LILACS, Scielo, and grey literature sources. Data were extracted and categorized according to the NREM domains: structure, process, and outcomes. **Results**: Thirty-two studies were included. Key barriers included limited AMS knowledge, role ambiguity, hierarchical dynamics, communication gaps, and lack of standardized nursing outcomes. Facilitators encompassed targeted AMS education, participation in multidisciplinary discussions, managerial support, and defined nursing roles. Nurse-led interventions showed potential to improve infection control and antibiotic administration, although standardized outcome reporting remains scarce. **Conclusions**: Framed by the NREM, this review underscores the essential contribution of nurses to AMS. Addressing structural barriers, enhancing role clarity, and fostering interdisciplinary collaboration are critical to enabling nurses’ full participation. Strengthening nursing engagement in AMS not only supports effective antimicrobial use and patient safety but also reinforces health system resilience and sustainability.

## 1. Introduction

The emergence of antimicrobial resistance (AMR) and the need to overcome this major problem has led to an increasingly broad perspective involving different stakeholders. AMR is defined as the resistance of bacterial, viral, parasitic, and fungal microorganisms to antimicrobial medicines that were previously effective for the treatment of infections. AMR occurs naturally over time but is accelerated by the inappropriate use of antimicrobials, which has led to this public health problem, also known as a silent pandemic [1]. The World Health Organization underscores the necessity for a coordinated global effort to enhance the understanding of AMR, emphasizing the importance of robust epidemiological surveillance and the implementation of strategies such as antimicrobial stewardship programmes to optimize the use of antimicrobials [1]. Aiming to tackle AMR, antimicrobial stewardship (AMS) is a broad term used to describe a coherent set of actions that promote the responsible use of antimicrobials, also referred to as responsible use, judicious use, or optimal use [2]. AMS is a coordinated program to promote the appropriate use of antimicrobial agents, leading to improved outcomes for citizens and healthcare systems. Although these programmes primarily focus on antimicrobial prescriptions, this emphasis may inadvertently overlook or undervalue the roles of other key stakeholders in the antimicrobials use cycle [2], particularly nurses. Within healthcare structures and processes, nurses are responsible for providing health promotion, education, and coordinating patient care in collaboration with a wide range of other healthcare professionals [3,4]. Nurses also play a crucial role in regulating the use of antimicrobials, administering medication, and monitoring drugs [5]. Therefore, several nursing interventions overlap with AMS objectives and clearly align with Irvine’s Nursing Role Effectiveness Model (NREM) [3], which discusses specific relationships between healthcare structural variables and nurses’ role functions or processes as well as their impact on patient and system outcomes. The Centers for Disease Prevention and Control (CDC) has updated the Core Elements of Hospital Antibiotic Stewardship Programs to include a new category of nursing-based interventions [6]. This update recognises the pivotal role of nurses in implementing key stewardship activities, including optimising microbiology cultures, facilitating intravenous to oral transitions, and prompting antibiotic reviews—“timeouts”. Furthermore, the CDC emphasises the importance of support from hospital leadership, particularly from chief nursing officers, to empower nurses in these roles [7]. This finding aligns with the Nursing Role Effectiveness Model’s emphasis on structural empowerment, suggesting that when nurses are supported by leadership and provided with the necessary resources, they can significantly impact patient care outcomes through effective antibiotic stewardship practices [3]. The Australian Commission on Safety and Quality in Health Care recommends formally including nurses in both the Antimicrobial Stewardship Committee and the Antimicrobial Stewardship Team, providing guidance on the role of nurses, midwives, and infection control practitioners [6,7]. These additions reflect the important role that nurses play in hospital AMS efforts. Despite growing calls for the formal recognition of nurses’ contributions, their involvement in AMS programmes remains limited [8,9]. As stated by Olans et al., nurses’ participation in AMS is not intended to overcome other healthcare professionals’ responsibilities, such as prescription, where appropriate [5]. Rather, it seeks to acknowledge and strengthen existing nursing interventions, aligning them more closely with optimal AMS goals and outcomes. Importantly, both patient and organizational outcomes are influenced by nursing-sensitive outcomes. These reflect the quality and effectiveness of nursing interventions and include clinical indicators, functional status improvement, patient experience measures, and broader health system outcomes [10].

Previous studies have primarily focused on exploring nurses’ perceptions of barriers to their participation in AMS programmes [11,12,13] as well as their competencies and contributions to these initiatives [9,14,15]. However, much of this research is context-specific and may not be generalizable across international settings due to variations in regulatory frameworks and clinical practices. A recent review further explored the role of nurses in facilitating change by identifying barriers and enablers, but it was limited to qualitative studies published over a six-year period [16]. As a result, existing reviews may not fully capture the broader spectrum of challenges, opportunities, and outcomes related to nurses’ engagement in AMS.

An initial search of MEDLINE, CINAHL, the Cochrane Database of Systematic Reviews, PROSPERO, and Joanna Briggs Institute (JBI) evidence synthesis revealed that, to the best of our knowledge, no prior review has offered a synthesized and theory-driven analysis on the structural and process-related factors influencing nurses’ participation in AMS programmes globally, particularly those that may affect nursing-sensitive outcomes. Therefore, this mapping review aims to identify the barriers and facilitators reported to influence nurses’ engagement in AMS programmes. Additionally, it seeks to identify nursing-sensitive outcomes related to nurses’ involvement in AMS.

## 2. Materials and Methods

A mapping review was selected over a scoping review to enable a broader, theory-informed synthesis of the literature, aimed at identifying the range, characteristics, and contextual variables, particularly structural and process-related factors, influencing nurses’ engagement in AMS programmes, as consistent with the objectives outlined by Campbell et al. [17]. The Preferred Reporting Items for Systematic Reviews and Meta-Analyses extension for Scoping Reviews (PRISMA-ScR) checklist was used to report this review [18]. The review protocol was previously registered on Open Science Framework https://doi.org/10.17605/OSF.IO/TN46C (accessed on 28 February 2024).

### 2.1. Eligibility Criteria

According to the JBI guidelines, inclusion and exclusion criteria were outlined for each component of the PCC framework—Population, Concept, and Context—to ensure a systematic and transparent selection process in the review [19]. Concerning the population, studies that included registered nurses working in healthcare settings were deemed eligible. Where evidence incorporates the analysis of other healthcare practitioners such as physicians and pharmacists, only data pertaining to nurses were extracted. Studies focusing on nursing students, dental nurses, veterinary nurses, and nursing assistants were also excluded to mitigate interpretation bias, considering the heterogeneity of these roles across international settings. Barriers were considered as any circumstances or behaviours that hinder nurses’ engagement in AMS, while facilitators were considered as any circumstances that enable nurses’ interventions in AMS. As the context of this review, we considered studies that report on healthcare provision taking place at any level (e.g., primary care or tertiary care), whether in public or private institutions.

Only findings written in English, Spanish, and Portuguese were deemed eligible.

### 2.2. Types of Sources

The review considered all type of primary studies that met the eligibility criteria. In addition, grey literature relevant to the topic was eligible, such as dissertations and technical reports. Expert opinion sources, including literature reviews, commentaries, and all types of systematic reviews that meet the inclusion criteria were also included. All international evidence of relevance from any geographic location was considered for inclusion in this review.

### 2.3. Search Strategy

The search strategy followed three stages: (i) a search of MEDLINE (via PubMed) and CINAHL (via EBSCOhost) to identify keywords, including descriptors and natural language, from terms used in titles, abstracts, and index terms for conjugation of indexing terms and words according to each database for subsequent searches; (ii) searches across MEDLINE (via PubMed), Web of Science, Scielo, CINAHL (via EBSCOhost), Nursing & Allied Health Collection (via EBSCOhost), Cochrane Plus Collection (via EBSCOhost), MedicLatina (via EBSCOhost), and the Open Access Scientific Repositories of Portugal (RCAAP) for unpublished studies; (iii) the reference list of all included sources of evidence, which was screened for additional studies. The search was conducted in November 2023. The search strategy is outlined in Table 1.

### 2.4. Source of Evidence Selection

**Figure 1:**
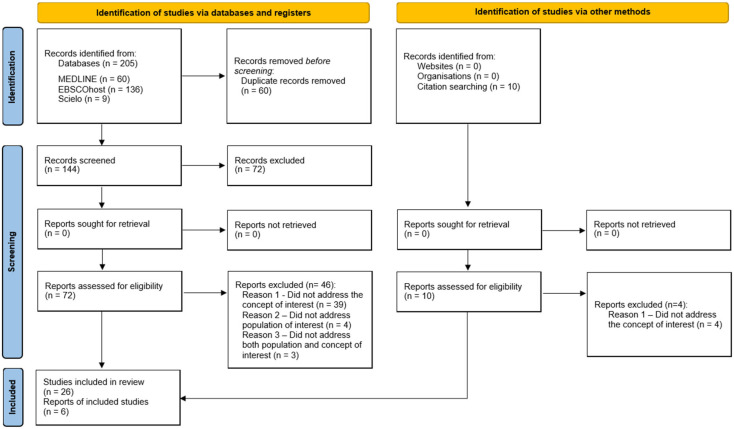
Search results, source selection, and inclusion process [20].

Following the search, duplicates were automatically removed in EBSCOhost search, and all identified papers were uploaded to Microsoft 365 One Drive, where duplicates were manually removed. Two independent reviewers (S.F. and P.S.C.) screened the titles and abstracts against the inclusion criteria. Any disagreements about the eligibility of a paper were resolved with an additional reviewer (A.C.). Then, the two reviewers screened the full texts of the articles against the inclusion criteria, and again, any disagreements about the eligibility of a paper were resolved through consultation of a third reviewer (A.C.). The Preferred Reporting Items for Systematic Reviews and Meta-Analyses (PRISMA) flow diagram (Figure 1) shows the search strategy and study inclusion process. The decisions for exclusion are reported according to PRISMA-ScR [18].

### 2.5. Data Extraction

Data were independently extracted by two reviewers (S.F. and P.C.S.) using a data extraction instrument developed by the research team, as described in the study protocol (in Supplementary Materials—Table S1: Data Extraction Instrument). The instrument was structured according to the theoretical components of the Nursing Role Effectiveness Model [3]. Extracted data included (1) title; (2) first author; (3) year of publication; (4) country of origin; (5) study design; (6) study objectives; (7) type of nurses participation in the study; (8) identification of barriers; (9) identification of facilitators; (10) nursing-sensitive outcomes. The reviewers used Microsoft 365 Excel Sheets to manage the extracted data. Any disagreements that arose between reviewers (S.F. and P.S.C.) were resolved through discussion or with a third reviewer (A.C.).

### 2.6. Data Analysis and Presentation

The final report followed the PRISMA-ScR guidelines [18]. A narrative summary describes how the results relate to the review’s objective. The full results of the search are presented in table format, aligned with the objective and review question (in Supplementary Materials—Table S2: Data extraction and included studies synthesis).

## 3. Results

A total of 205 records were retrieved through the search strategy. From these, 60 duplicates were removed (42 automatically removed in EBSCOhost search and 12 manually identified and removed). The reviewers then screened 144 titles and abstracts against the inclusion criteria. Of the potentially relevant reports, we sought 72 full-text citations, which were assessed in detail against the inclusion criteria. The reasons for excluding 46 full-text papers are provided in Supplementary Materials—Table S3: Excluded papers. Hand searches of relevant reviews and the reference lists of included sources produced an additional 10 articles, from which 4 were excluded because they did not address the concept of interest. A total of 32 sources were included in this scoping review. The search results, source selection, and inclusion process are presented in Figure 1.

### 3.1. Characteristics of Included Sources

A total of 32 studies published between 2014 and 2023 were included in this review, the majority of which were descriptive in nature. The qualitative studies (*n* = 15) encompassed descriptive studies with semi-structured interviews (*n* = 9), exploratory studies with semi-structured interviews (*n* = 4), a descriptive study with in-depth interviews (*n* = 1), and a prospective descriptive study with semi-structured interviews (*n* = 1). The quantitative studies (*n* = 8) comprised quasi-experimental studies (*n* = 2), descriptive cross-sectional studies (*n* = 3), a single-centre cross-sectional study (*n* = 1), and descriptive studies (*n* = 3). In addition, three studies utilising a mixed-methods approach were incorporated into the analysis. Moreover, the review encompassed a range of literature reviews, including integrative literature reviews (*n* = 3), scoping reviews (*n* = 2), and a single narrative literature review (*n* = 1).

Excluding the reviews, most of the studies were conducted in hospitals (*n* = 28), and one was conducted in residential aged care facilities (*n* = 1).

Studies reporting on barriers and facilitators to nurses’ engagement in AMS were conducted in Australia (*n* = 8), the United States (*n* = 7), the United Kingdom (*n* = 5), and Canada (*n* = 3). One study was identified from each of the following countries: Malaysia, Egypt, Singapore, Malawi, Pakistan, South Africa, Thailand, Norway, and China.

**Figure 2:**
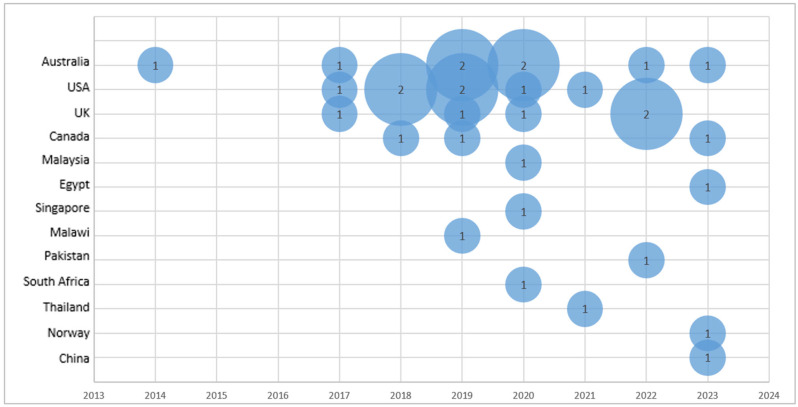
Bubble plot of the articles included in the review by year and author-affiliated country (the number inside the bubble corresponds to the number of articles published in that year in the specified country).

The distribution of studies by year and country is presented in Figure 2.

The 12 studies that exclusively involved nurses [12,21,22,23,24,25,26,27,28,29,30,31] had varying numbers of participants, ranging from 15 [12] to 583 [27]. For the remaining empirical articles, the number of nurse participants with other healthcare professionals ranged from 7 in 9 [32] to 40 in 61 [33]. Other health professionals frequently included in the studies population were physicians and pharmacists [13,32,33,34,35,36,37,38,39,40,41,42]; however, the data retrieved from these studies were only the characteristics of interest to nurses, as explained in eligibility criteria.

Data extracted from the included papers are presented in Supplementary Materials—Table S2: Data extraction and included studies synthesis. While all included studies (*n* = 32) described at least one barrier to nurses’ engagement in AMS programmes, facilitators to this role were described in most of the included studies (*n* = 27, 84.38%).

### 3.2. Barriers to Nurses’ Engagement in AMS Programmes

The literature identified several barriers to nurses’ engagement in AMS, including patient/family, individual, team, organizational, and regulatory factors.

Patient/family expectations such as misconceptions on antibiotic treatment, poor adherence to antibiotic regimens, and a lack of trust in nurses’ advice [31,39,43] were perceived as challenges to nurses’ role in delivering educational interventions within AMS.

Within the individual scope, gaps in knowledge and education related to AMR and AMS [9,12,13,15,21,23,24,25,29,30,31,32,33,34,35,37,41,42,44,45,46,47], particularly concerning the concept of AMS, AMS programmes, principles of antimicrobials use [22,23,24,27,28,29,30,35,38,40,44], and the impact of inappropriate specimen culturing techniques [21], were identified as barriers. These gaps may lead to low awareness of the AMS concept [22,31,32,33,35,37,39,41,46,48,49,50]. Nurses’ low motivation was also reported as a constraint [38]. Additionally, some nurses believed that their interventions were not applicable to AMS [12,15,21,25,32,37,38,41,42], failing to recognize their daily practices’ contributions [38,47].

Team-related barriers included professional hierarchies and boundaries [9,12,22,24,25,26,29,31,35,36,37,38,41,42,43,46,47,49], such as resistance from other healthcare professionals to nurses’ suggestions, sometimes described as pushback [9,21,27,28,33,44,47,49]. Defective communication between professionals [22,23,24,27,39,44] and non-inclusive antimicrobial discussions [9,22,24,26,27,28,34,35,37,45,46] were also considered to hinder nurses’ inclusion in AMS.

At the organizational level, lack of support from management [26,28,38,41] and the absence of standardized procedures or guidelines [12,24,40,44] were identified as key challenges. At the regulatory level, the lack of a clearly defined role for nurses in AMS [9,15,21,24,26,29,38,40,43] contributed to the under-recognition of nurses’ contributions among peers and within the multidisciplinary team. Additional barriers included increased workload [23,32,33,39,44,46,49] and lack of resources [22,38,39,45], such as nursing staff shortage, lack of experienced nurses, high staff rotation and turnover, and inadequate opportunities for in-service training. These constraints may lead nurses to prioritize other aspects of care, perceiving AMS as lower priority [12,25,39]. Likewise, at the point of care transfer, inaccessibility of information at the organizational or system level was identified as a significant barrier to AMS [34,44]; specifically within the emergency department, the pressure to transfer patients to the ward was associated with delays in antimicrobials administration [44].

Moreover, inadequate information technology systems [40,42] were also identified as a constraint. Although less frequently reported, the lack of nursing-sensitive outcomes in this field was also considered another barrier [21].

**Figure 3:**
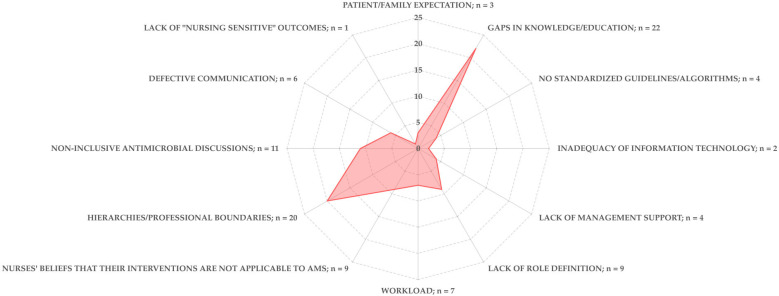
Radar graphic of the identified barriers to nurses’ engagement in AMS in the articles included in the review (where the fulfilment line crosses the radar line represents the number of times the barrier was identified).

The distribution of the identified barriers to nurses’ engagement in AMS is presented in Figure 3.

### 3.3. Facilitators to Nurses’ Engagement in AMS Programmes

The literature identified several facilitators to nurses’ engagement in AMS, spanning patient/family, individual, team, organizational, and regulatory domains.

Patients and caregivers/family literacy was found to support responsible antibiotic use, as more informed individuals are more likely to express concerns about the antibiotics they receive [31].

Education on AMS and principles of antibiotic therapy is a key facilitator [9,12,13,22,23,24,25,26,33,38,41,43,44,45,46,47,49,50], with two studies highlighting the added value of integrating behaviour change strategies into educational interventions [46,49]. Harnessing nurses’ motivation as patient advocates [9,12,15,22,26,29,35,46,47,49] can further enhance public awareness of appropriate antibiotic use and simultaneously elevate the nursing role in AMS efforts [31]. The perception that nurses do already contribute meaningfully to AMS [21,29,31,35,38,41,46,50] along with their constant presence at the patient’s bedside [12,21,46,49] and across the care continuum [45] is as important enabler of their engagement.

Other facilitators include participation in ward round discussions [12,24,25,28,29,31,32,46,49], involvement in multidisciplinary antibiotic decision making [9,13,31,32,35,38,40,42,49], status as local AMS champions [15,32,40,42,43], and the implementation of effective communication strategies [9,15,22,25,32,44,46,50]. The use of improvement models and measurement of the outcomes [25] have also been recognized as beneficial.

At the organizational level, key facilitators include the development of guidelines or algorithms to support decision making and communication with peers or other healthcare professionals [9,12,21,25,31,32,33,38,44,46], support from management [23,31,41,43,47,50], and nurses’ formal inclusion in AMS programmes [13,24,49]. Additionally, optimizing information technologies [49] offers further opportunities to enhance nurses’ participation.

A clearly defined nursing role within AMS [13] may foster recognition of independent nursing interventions by the multidisciplinary AMS team, thereby strengthening nurses’ engagement [29].

**Figure 4:**
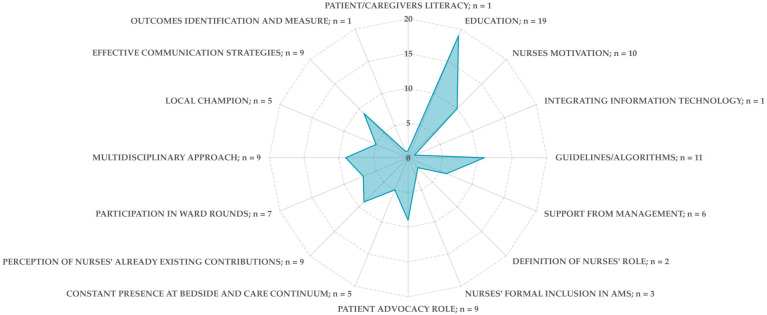
Radar graphic of the identified facilitators to nurses’ engagement in AMS in the articles included in the review (where the fulfilment line crosses the radar line represents the number of times the barrier was identified).

Figure 4 presents an overview of the identified facilitators.

### 3.4. Outcomes Associated with Nurses Engagement in AMS

The identification of outcomes associated with nurses’ engagement in AMS emerged as a secondary objective of this review, which retrieved limited evidence on the topic. Nonetheless, potential outcomes were identified across several domains, including education, microbiological cultures, antibiotic administration, device-associated infections, and allergy documentation.

Some studies highlighted specific improvements linked to nurse-led interventions. These include the prevention of device-associated infections such as central-line-associated bloodstream infections and catheter-associated urinary tract infections [15], enhanced antibiotic prophylaxis timing [38], and increased knowledge regarding adverse effects of practices related to antibiotic preparation [23].

Educational interventions targeting nurses were primarily associated with improvements in urine culture practices [9,15,21,49] and in the transition from intravenous (IV) to oral antibiotic therapy [9,42,48,49]. These initiatives were linked to enhanced nurse confidence and a greater willingness to question antibiotic prescriptions or advocate for appropriate IV to oral switch. This, in turn, may contribute to reduced antibiotic use [49] and shorter hospital stays [48]. However, no studies reported direct patient or organizational outcomes.

Antibiotic administration, a core component of nursing practice, is cited as a key area of involvement in AMS [15,49,50]. Improved adherence to best practice through a reduction in medication errors [22,50] and better timing of surgical antibiotic prophylaxis [38] were reported as potential outcomes.

Although patient education was recognized as an important nursing intervention within AMS programmes [42,49], no specific outcomes related to education initiatives were identified in this review. Similarly, while allergies identification and documentation were acknowledged as a critical nursing responsibility in AMS [38,42], direct outcomes linked to these activities were not reported.

## 4. Discussion

This mapping review aimed to identify barriers and facilitators influencing nurses’ engagement in AMS programmes and identifies critical factors influencing nurses’ participation in AMS programs, addressing individual, team-based, organizational, and regulatory challenges across the various international settings represented in this review. Across the reviewed studies, several recurrent themes emerged, including structural and organizational challenges such as insufficient education, limited role clarity, and professional boundaries. Facilitators were also identified, notably targeted training initiatives, managerial endorsement, and interdisciplinary collaboration, all of which contributed to enhancing nurses’ meaningful participation in AMS. A secondary objective of this review was to explore nursing-sensitive outcomes associated with such engagement. Although few studies addressed this dimension directly, some reported outcomes, such as improved device-associated infection rates and antibiotic prophylaxis timing, suggest that nursing interventions can positively influence both clinical effectiveness and patient safety. Notably, our findings show a consistent interplay between structural conditions, nursing processes, and the observable outcomes, demonstrating the Nursing Role Effectiveness Models’ core premise that nursing effectiveness arises from this dynamic interdependence.

According to Irvine et al.’s NREM [3], nursing care generates outcomes within a multifactorial system that interconnects structural factors—including variables related to nurses, patients, and healthcare organizations—and process factors—which refer to the nursing role expressed through independent, dependent, and interdependent actions [3]. The authors emphasize that nurses hold a privileged role in overseeing healthcare delivery and that nursing-sensitive outcomes are shaped by both structural and process-related factors.

**Figure 5:**
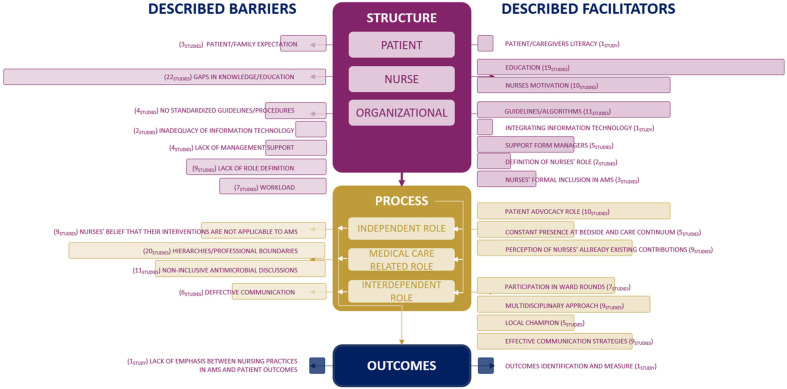
The central element of this figure is a free adaptation of Irvine et al.’s NREM [3], which offers an overview of the interconnection between the model domains and the identified variables in this review, combined in bar graphs.

To guide our discussion, we synthetized the results of this review according to the domains of the NREM (Figure 5), which offers a theoretical lens for understanding how structural and process-related factors influence nursing-sensitive outcomes. By mapping identified barriers and facilitators to the model’s domains, this synthesis highlights the complex interplay between structural and process domains that shape nurses’ participation in AMS and ultimately impact outcomes. From the synthesis presented in Figure 5, several key themes emerge, which are discussed in detail in the following sections.

### 4.1. Structure Domain

The structure domain encompasses variables related to patients, nurses, and organizational factors that influence the processes and outcomes of care [10]. Patient-related structural variables include attributes such as age, physical functioning, and the presence of comorbidities, which can affect patients’ care needs and outcomes. Nurse-related structural variables comprise factors like experience level, knowledge base, and skill proficiency, all of which contribute to the nurse’s capacity to provide effective care. Organizational structural variables focus on aspects such as staffing patterns, including nurse-to-patient ratios and skill mix, which are expected to impact care quality. Structural measures assess the capacity to provide care but do not directly evaluate the care itself. Therefore, while deficiencies in structural variables may indicate potential issues, they are not definitive evidence of poor care or outcomes [10].

Within this domain, education emerged as a central factor influencing nurses’ engagement in AMS. Gaps in knowledge and education were among the most frequently reported barriers to nurse engagement in AMS, while structured educational interventions were consistently identified as key enablers. These findings underscore the critical need for ongoing professional development initiatives, constant feedback on surveillance data, and other indicators that foster critical thinking and embed stewardship principles into nursing education. This is supported by Chater et al., who reported significantly higher AMS-related competencies, including behaviours, skills, and self-perceived capability to engage in AMS, among nurses who received training compared to those who did not [51]. Similarly, other studies observed a positive correlation between targeted education and improvements in nurses’ knowledge, awareness, and attitudes towards AMS practices [23,47,52]. These educational improvements, however, invite further reflection on their practical implications. It is important to consider how enhanced knowledge and skills among nurses translate into tangible benefits for patient safety, clinical decision making, and professional accountability within AMS. Making this connection visible not only reinforces the value of educational interventions but also helps to articulate the distinct contribution of nursing practice to patient outcomes and wider organizational performance. Gillespie et al. offered a compelling example: in an educational intervention that emphasized the possibility of switching from intravenous (IV) to oral antibiotics, nurses were encouraged to consider the reasons for administering IV antibiotics, which was associated with an improvement in awareness of the risk of developing resistance, knowledge of the associated risk of line-related infection with intravenous therapy, and a decrease in line days [53]. This findings underscore the necessity for ongoing, structured educational programs tailored specifically to enhance nurses’ competencies in antimicrobial management, which was clearly demonstrated in Gillespie et al.’s study [53]. Addressing gaps in AMS education is necessary at both undergraduate and postgraduate levels despite ongoing efforts to bridge this gap [1]. Regulatory bodies play a critical role supporting and strengthening these efforts.

Role clarity and broader organizational support emerged in this review as key structural determinants influencing nurses’ engagement in AMS. While role definition is essential, it is equally important that nurses recognize and value their existing contributions. Nurses do already perform interventions that directly align with AMS objectives, including ensuring the quality of microbiological cultures, preventing device-associated infections, managing risk factors associated with invasive devices, preparing and administering antimicrobials, assessing allergies, and providing patient education [5]. The absence of a clearly defined role has been associated with limited accountability and participation, whereas formal recognition, through clinical guidelines, job descriptions, and performance evaluations, has been linked to greater engagement. Therefore, investing in outcome metrics and embedding these interventions within evidence-based frameworks can strengthen both professional accountability and the visibility of nursing contributions. As evidenced by Castro-Sánchez et al., integrating nursing responsibilities into protocols and interdisciplinary care models enhances both role clarity but also the overall impact of nurses in AMS initiatives [54]. Moreover, broader organizational facilitators, including leadership support, availability of clinical guidelines and algorithms, and the integration of supportive technologies, were linked to improved participation. The development of user-friendly platforms, integrating relevant up-to-date information to support antibiotic-related tasks, poses a significant opportunity improving accessibility to information and accuracy of care delivery, as recognized by Wentzel et al. [55].

In the daily routine of clinical practice, nurses do not always have the time or space to reflect on the broader impact of their professional actions, often perpetuating routines in an almost automatic manner. As highlighted in this review, challenges such as excessive workloads, high patient-to-nurse ratios, and understaffing are factors that, from our perspective, contribute to a less reflective practice and a more task-oriented one. Supporting nurses in linking their interventions to clinical outcomes and the broader challenge of AMR may foster critical thinking and promote the adoption of a more proactive and accountable role in AMS. Nonetheless, these initiatives must be supported by broader organizational strategies to ensure workforce stability. Challenges associated with rapid staff turnover, inexperienced personnel, and reliance on short-term contracts threaten the sustainability of AMS efforts; hence, the institution may never fully benefit from the investment made in training nurses. Such human resource strategies raise concerns about the feasibility of achieving meaningful clinical and organizational outcomes [54].

Although less commonly addressed, patient- and family-related variables also emerged as significant influences on nurses’ engagement in AMS. Within the NREM, nurse–patient–family relationships are understood not merely as interpersonal dynamics but as structural components that shape the delivery of care [10]. These dynamics directly impact nurses’ ability to implement AMS strategies, particularly patient education, communication, and advocacy. Patient education, a key independent nursing intervention recognized by the International Council of Nurses (ICN) [56], requires institutional support, interdisciplinary alignment and inclusive care protocols. Strengthening these organizational structures presents a significant opportunity to promote appropriate antimicrobial use such as adherence to treatment, enhance patient safety, and mitigate AMR. However, the mapped studies emphasize that such interventions remain underexplored, particularly in community care settings, where only one study was identified [33]. Enabling nurses to address health literacy and manage patient expectations as part of their independent scope is essential. Doing so not only aligns with the ICN’s call to optimize nursing roles in AMS but also expands stewardship efforts beyond acute care, fostering continuity and accessibility across healthcare systems [56].

### 4.2. Process Domain

Within this domain, our findings underscore the influence of nurses’ beliefs about their contributions to AMR mitigation as well as the impact of hierarchical dynamics and communication on their engagement.

Although the perception that their interventions lie outside the scope of AMS has been a recurring barrier, we argue that recognition of the nursing role must begin with nurses themselves. While the literature underscores role clarity and organizational support as key structural determinants of nurses’ engagement in AMS, self-awareness of their contributions to AMR mitigation and responsible antimicrobial use remains essential. Within the NREM framework, this aligns with the independent role of nurses, which encompasses activities such as patient assessment, monitoring and education, and infection prevention: interventions that directly influence antimicrobial use [10]. While organizational recognition, development of supportive guidelines, and clear definition of nursing responsibilities can encourage participation and enhance professional visibility, these measures may fall short if underlying beliefs persist that nursing practice has little impact on AMR. Thus, addressing internal perceptions is as crucial as institutional support to fully enabling the nursing contribution to AMS. Filipe et al. showed that targeted training can empower nurses to take greater ownership of their practice [57]. Their study reported a substantial reduction in blood culture contamination following a multimodal intervention, signalling a broader shift in accountability and stewardship culture. By reinforcing best practices and providing structural support, the intervention enabled nurses to act more consciously and effectively, demonstrating how education can drive meaningful and sustainable change in clinical behaviour. Such findings align with behaviour change theories like the Theory of Planned Behaviour, which suggests that meaningful engagement in new behaviours is shaped by individual attitudes, perceived social norms, and perceived behavioural control [58]. In the context of AMS, this underscores that nurses’ participation is strongly shaped by internal motivation and professional confidence—factors that can be strengthened through organizational structures that empower and legitimize nursing roles within stewardship initiatives.

While professional hierarchies and entrenched interprofessional boundaries were understood as barriers to nursing engagement in AMS, this review highlights a growing recognition of the essential role nurses play in antimicrobial use. As Broom et al. suggested, moving beyond rigid hierarchies toward shared accountability enables the integration of nursing perspectives and supports more holistic, patient-centred decision making [35]. It is therefore essential to align the responsibilities of all healthcare professionals in order to enhance nurses’ meaningful engagement in AMS.

Effective communication emerged as a cross-cutting theme and a fundamental mechanism for enabling inclusive and collaborative practice in this field. Our findings indicate that defective communication systems limited nurses’ opportunities to participate in stewardship activities, whereas structured, inclusive strategies significantly enhanced their involvement. Monsees et al. demonstrated how using communication tools like Situation-Background-Assessment-Recommendation (SBAR) facilitated interdisciplinary discussions around AMS, overcoming professional boundaries and focusing on patient care [25]. When successful multidisciplinary collaboration is developed and implemented, patient and organizational outcomes emerge, as demonstrated by Raybardhan et al.’s intervention [59]. Promoting active involvement of nurses on ward rounds, along with pharmacists and intensivists, resulted in a 20% reduction in antimicrobial use per month and an increase in the proportion of relevant cases for which an antimicrobial prompt was provided. Within this strategy, the development of a script to guide nurses facilitated communication. Additionally, Ha et al. found that nurse-driven rounds, attended by pharmacists and infection preventionists, led to reductions in antimicrobial use and urinary catheterization days [60]. These studies underscore the value of inclusive AMS frameworks that promote collaborative communication, shared decision making, and mutual recognition of professional contributions.

### 4.3. Outcomes

Our findings reveal a significant gap in the systematic measurement of AMS nursing contributions to patient outcomes. Only 9% of the studies included in this review evaluated the impact of nursing practices on clinical or antimicrobial outcomes [15,23,38]. This deficiency limits the ability to measure nurses’ direct contributions to AMS efforts and may hinder efforts to further integrate nursing perspectives. Organizational support is essential for any improvement project to prevail, but the lack of evidence may undermine strategic healthcare decision making towards antimicrobials preservation, as underlined by Pombo et al. [61].

The mapped studies highlighted opportunities to link nursing interventions to AMS and improved patient outcomes. For example, in studies reporting enhanced collection of biological specimens following targeted educational interventions for nurses, the authors suggest that these improvements may contribute to reduced antimicrobials use and shorter hospital stays [9,15,21,49]. However, based on our analysis, these outcomes often lack objective and measurable indicators that would help nurses understand the actual impact of their care on patient outcomes. For instance, reporting blood culture contamination rates before and after an educational intervention would provide a more precise and informative result [57].

Although the outcomes domain was the least represented, it remains critically important. Only one study explicitly examined the link between nursing practice and AMS outcomes [38], revealing a substantial gap in measurement and reporting. This lack of evidence may hinder the visibility and strategic advancement of nurse-led interventions within AMS.

Nursing outcomes are measurable changes in a patient’s health status, behaviour, or knowledge that can be directly linked to nursing interventions. They serve as key indicators of the quality and effectiveness of nursing care and are essential for assessing both individual performance and broader system-level impact [10]. Although challenging due to the interdependency of nursing interventions and the multifactorial contributions to the development of healthcare-associated infections and antimicrobial resistance, clearly isolating outcomes attributable solely to nursing can be challenging. As a result, many nursing-sensitive outcomes remain underreported or are embedded within broader healthcare indicators, potentially minimizing the visibility of nursing’s unique contribution. To address this, it is crucial to establish and adopt nursing-sensitive indicators that reflect both independent and collaborative actions. Within the context of AMS, relevant nursing outcomes may include reduced infection rates, improved patient adherence to treatment, and enhanced education regarding appropriate antimicrobial use as reported in the literature [15,42,49]. Other critical indicators include improved diagnostic accuracy (reducing misclassification of colonization or asymptomatic bacteriuria as infection and reducing blood and urine culture contamination rates), the number of catheterization days (urinary and peripheral-inserted venous catheters), and the rate of intravenous-to-oral antimicrobial conversion [9,12,21,49]. Antimicrobials administration is a core nursing responsibility and a critical component ensuring the efficacy of antimicrobial therapy. However, we identified only three references to this intervention in the literature, and no nursing-sensitive outcomes were reported [15,49,50]. This represents a significant gap and highlights a key area for further investment and research, as also noted by Van Huizen [50]. Additionally, length of patient stay and economic impact study on nursing outcomes could provide deeper insights into the overall impact of nursing within AMS programs. Finally, although it is more challenging to measure, as it depends on sustainability, changes in antimicrobial resistance patterns could provide insight in further study.

Future research should prioritize rigorous outcome measurement to more robustly substantiate the role of nurses in achieving AMS objectives. Strengthening the evidence base through structured data collection and the consistent use of validated metrics will enhance accountability, support professional recognition, and promote the effective integration of nursing within AMS programs. To advance this agenda, it is essential to establish international consensus on the definition and operationalization of key indicators as well as to develop standardized instruments that facilitate data collection, systematic evaluation, and benchmarking across healthcare organizations.

Particular attention should be given to evaluating nursing interventions and expanding research within community care settings—such as primary care and residential aged care homes—where evidence remains notably scarce. This scarcity is underscored by our review, which identified only one study conducted in a residential aged care home [33].

### 4.4. Strengths and Limitations

Our review’s findings must be interpreted considering its methodological limitations. Regarding the strengths of this review, we adhered to the recommendations provided by JBI for “big picture” reviews, opting for the systematic mapping review approach as the most appropriate method to address our initial research question [17]. To mitigate potential bias, this review was conducted based on a pre-registered protocol publicly available. Furthermore, data extraction and analysis were conducted following the core dimensions of a well-established nursing model.

However, it is essential to acknowledge certain limitations that should be considered when discussing our findings. Firstly, while not obligatory for mapping reviews, we chose not to include or exclude potential findings based on their methodological quality. Secondly, the review process aimed to strike a balance between search specificity and sensitivity by developing a comprehensive strategy incorporating various natural language terms and descriptors commonly used in this field, tailored to the most frequently used databases and repositories in nursing science. However, as with any attempt to synthesize evidence, there is a possibility that relevant studies and data may have been inadvertently excluded. Additionally, considering the research team’s expertise in infection control and AMS, we acknowledge the potential for confirmation bias during data analysis, interpretation, and synthesis.

## 5. Conclusions

Framed by the NREM, the findings of this review reinforce the central role of nurses in AMS and highlight key areas for future research, implementation, and policy development. The analysis demonstrates that structural conditions, such as institutional support and clear role definitions, are closely interwoven with nursing processes, including clinical decision making, interdisciplinary communication, and education. Together, these elements contribute to measurable outcomes in patient care and system performance, reflecting the core premise of the NREM: that nursing effectiveness emerges from the dynamic interaction between structure, process, and outcomes. Advancing nurses’ meaningful engagement in AMS therefore requires addressing the domains not in isolation but as mutually reinforcing components of care.

This review also identifies significant barriers to nurse engagement, including gaps in education, limited role clarity, restrictive professional boundaries, and a lack of recognition of nursing’s relevance to AMS. However, where barriers exist, opportunities also emerge. Facilitators such as targeted training, professional motivation, inclusive decision making, and strong organizational support offer tangible pathways to enhance nursing contributions to stewardship efforts.

To ensure the effective integration of nurses into AMS programs, healthcare systems must adopt multifaceted strategies that encompass comprehensive education, clear and expanded nursing roles, inclusive team dynamics, and robust communication infrastructures. Addressing these factors has the potential not only to strengthen existing AMS initiatives but also improve patient safety, optimize antimicrobial use, and enhance overall system resilience.

## Figures and Tables

**Table 1:** Search strategy (conducted in November 2023).

Database	Search	Results Retrieved
MEDLINE via PubMed	((Nurses) AND (antimicrobial stewardship) AND (acute care OR healthcare)) AND ((facilitators AND barriers) OR (perception*))	60
EBSCOhost Research	(“antimicrobial stewardship” AND nurs*) AND engage* OR (“nurs* perception*” AND “antimicrobial stewardship”) OR “nurs* behaviour*” AND “nurs* role” AND (antibiotic* OR antimicrob*) NOT (patient* OR psyc* OR veterin* OR dent* OR disinfect*) AND (barrier* and facilitator* or enabler*)	136
Scielo	(*Antibiotic OR Antimicrobial) AND (stewardship OR management) AND (nurses OR nurse OR nursing) AND NOT (veterinary)	9

## Data Availability

The original contributions presented in the study are included in the article/Supplementary Materials, and further inquiries can be directed to the corresponding author.

## Supplementary Materials

The following supporting information can be downloaded at https://www.mdpi.com/article/10.3390/nursrep15060216/s1. Table S1: Data extraction instrument; Table S2: Data extraction and included studies synthesis; Table S3: Full-text studies that did not meet the inclusion criteria.

## Public Involvement Statement

There was no public involvement in any aspect of this research.

## Guidelines and Standards Statement

This manuscript was drafted against the PRISMA Extension for Scoping Reviews (PRISMA-ScR) guidelines [18].

## References

[1] WHO (2017). Fact Sheet on Sustainable Development Goals (SDGs): Health Targets—Antimicrobial Resistance.

[2] Dyar O.J., Huttner B., Schouten J., Pulcini C. (2017). What Is Antimicrobial Stewardship?. Clin. Microbiol. Infect..

[3] Irvine D., Sidani S., Hall L.M. (1998). Finding Value in Nursing Care: A Framework for Quality Improvement and Clinical Evaluation. Nurs. Econ..

[4] Sumner S., Forsyth S., Collette-Merrill K., Taylor C., Vento T., Veillette J., Webb B. (2018). Antibiotic Stewardship: The Role of Clinical Nurses and Nurse Educators. Nurse Educ. Today.

[5] Olans R.N., Olans R.D., DeMaria A. (2016). The Critical Role of the Staff Nurse in Antimicrobial Stewardship—Unrecognized, but Already There. Clin. Infect. Dis..

[6] CDC (2019). The Core Elements of Hospital Antibiotic Stewardship Programs.

[7] (2018). Australian Comission on Safety and Quality in Health Care. Antimicrobial Stewardship in Australian Health Care.

[8] ANA/CDC (2017). Redefining the Antibiotic Stewardship Team: Recommendations from the American Nurses Association/Centers for Disease Control and Prevention Workgroup on the Role of Registered Nurses in Hospital Antibiotic Stewardship Practices. JAC-Antimicrob. Resist..

[9] Davey K., Aveyard H. (2022). Nurses’ Perceptions of Their Role in Antimicrobial Stewardship within the Hospital Environment. An Integrative Literature Review. J. Clin. Nurs..

[10] Irvine D., Sidani S., Hall L.M. (1998). Linking Outcomes to Nurses’ Roles in Health Care. Nurs. Econ..

[11] Abbas S., Lee K., Pakyz A., Markley D., Cooper K., Vanhoozer G., Doll M., Bearman G., Stevens M.P. (2019). Knowledge, Attitudes, and Practices of Bedside Nursing Staff Regarding Antibiotic Stewardship: A Cross-Sectional Study. Am. J. Infect. Control.

[12] Fisher C.C., Cox V.C., Gorman S.K., Lesko N., Holdsworth K., Delaney N., McKenna C. (2018). A Theory-Informed Assessment of the Barriers and Facilitators to Nurse-Driven Antimicrobial Stewardship. Am. J. Infect. Control.

[13] Rout J., Brysiewicz P. (2020). Perceived Barriers to the Development of the Antimicrobial Stewardship Role of the Nurse in Intensive Care: Views of Healthcare Professionals. S. Afr. J. Crit. Care.

[14] Danielis M., Regano D., Castaldo A., Mongardi M., Buttiron Webber T. (2022). What Are the Nursing Competencies Related to Antimicrobial Stewardship and How They Have Been Assessed? Results from an Integrative Rapid Review. Antimicrob. Resist. Infect. Control.

[15] Zhao W., Guo W., Sun P., Yang Y., Ning Y., Liu R., Xu Y., Li S., Shang L. (2023). Bedside Nurses’ Antimicrobial Stewardship Practice Scope and Competencies in Acute Hospital Settings: A Scoping Review. J. Clin. Nurs..

[16] Bonacaro A., Solfrizzo F.G., Regano D., Negrello F., Domeniconi C., Volpon A., Taurchini S., Toselli P., Baesti C. (2024). Antimicrobial Stewardship in Healthcare: Exploring the Role of Nurses in Promoting Change, Identifying Barrier Elements and Facilitators—A Meta-Synthesis. Healthcare.

[17] Campbell F., Tricco A.C., Munn Z., Pollock D., Saran A., Sutton A., White H., Khalil H. (2023). Mapping Reviews, Scoping Reviews, and Evidence and Gap Maps (EGMs): The Same but Different—the “Big Picture” Review Family. Syst. Rev..

[18] Tricco A.C., Lillie E., Zarin W., O’Brien K.K., Colquhoun H., Levac D., Moher D., Peters M.D.J., Horsley T., Weeks L. (2018). PRISMA Extension for Scoping Reviews (PRISMA-ScR): Checklist and Explanation. Ann. Intern. Med..

[19] Peters M.D.J., Godfrey C., McInerney P., Khalil H., Larsen P., Marnie C., Pollock D., Tricco A.C., Munn Z. (2022). Best Practice Guidance and Reporting Items for the Development of Scoping Review Protocols. JBI Evid. Synth..

[20] Page M.J., McKenzie J.E., Bossuyt P.M., Boutron I., Hoffmann T.C., Mulrow C.D., Shamseer L., Tetzlaff J.M., Akl E.A., Brennan S.E. (2021). The PRISMA 2020 Statement: An Updated Guideline for Reporting Systematic Reviews. BMJ.

[21] Carter E.J., Greendyke W.G., Furuya E.Y., Srinivasan A., Shelley A.N., Bothra A., Saiman L., Larson E.L. (2018). Exploring the Nurses’ Role in Antibiotic Stewardship: A Multisite Qualitative Study of Nurses and Infection Preventionists. Am. J. Infect. Control.

[22] Hamdy R.F., Neal W., Nicholson L., Ansusinha E., King S. (2019). Pediatric Nurses’ Perceptions of Their Role in Antimicrobial Stewardship: A Focus Group Study. J. Pediatr. Nurs..

[23] Hendy A., Al-Sharkawi S., Hassanein S.M.A., Soliman S.M. (2023). Effect of Educational Intervention on Nurses’ Perception and Practice of Antimicrobial Stewardship Programs. Am. J. Infect. Control.

[24] Monsees E., Popejoy L., Jackson M.A., Lee B., Goldman J. (2018). Integrating Staff Nurses in Antibiotic Stewardship: Opportunities and Barriers. Am. J. Infect. Control.

[25] Monsees E., Lee B., Wirtz A., Goldman J. (2020). Implementation of a Nurse-Driven Antibiotic Engagement Tool in 3 Hospitals. Am. J. Infect. Control.

[26] Mostaghim M., Snelling T., McMullan B.J., Konecny P. (2017). Nurses Are Underutilised in Antimicrobial Stewardship e Results of a Multisite Survey in Paediatric and Adult Hospitals. Infect. Dis. Health.

[27] Mustafa Z.U., Manzoor M.N., Shahid A., Salman M., Hayat K., Yasmin K., Baraka M.A., Mathew S., Kanwal M., Parveen S. (2022). Nurses’ Perceptions, Involvement, Confidence and Perceived Barriers Towards Antimicrobial Stewardship Program in Pakistan: Findings from a Multi-Center, Cross-Sectional Study. J. Multidiscip. Healthc..

[28] Padigos J., Reid S., Kirby E., Anstey C., Broom J. (2023). Nursing Experiences in Antimicrobial Optimisation in the Intensive Care Unit: A Convergent Analysis of a National Survey. Aust. Crit. Care.

[29] Tangeraas Hansen M.J., Storm M., Syre H., Dalen I., Husebø A.M.L. (2023). Attitudes and Self-Efficacy towards Infection Prevention and Control and Antibiotic Stewardship among Nurses: A Mixed-Methods Study. J. Clin. Nurs..

[30] Wilcock M., Harding G., Moore L., Nicholls I., Powell N., Stratton J. (2013). What Do Hospital Staff in the UK Think Are the Causes of Penicillin Medication Errors?. Int. J. Clin. Pharm..

[31] Wong L.H., Bin Ibrahim M.A., Guo H., Kwa A.L.H., Lum L.H.W., Ng T.M., Chung J.S., Somani J., Lye D.C.B., Chow A. (2020). Empowerment of Nurses in Antibiotic Stewardship: A Social Ecological Qualitative Analysis. J. Hosp. Infect..

[32] Groumoutis J.Y., Gorman S.K., Beach J.E. (2023). Identifying Opportunities for Antimicrobial Stewardship in a Tertiary Intensive Care Unit: A Qualitative Study. Can. J. Crit. Care Nurs..

[33] Lim C.J., Kwong M., Stuart R.L., Buising K.L., Friedman N.D., Bennett N., Cheng A.C., Peleg A.Y., Marshall C., Kong D.C. (2014). Antimicrobial Stewardship in Residential Aged Care Facilities: Need and Readiness Assessment. BMC Infect. Dis..

[34] Black E.K., MacDonald L., Neville H.L., Abbass K., Slayter K., Johnston L., Sketris I. (2019). Health Care Providers’ Perceptions of Antimicrobial Use and Stewardship at Acute Care Hospitals in Nova Scotia. Can. J. Hosp. Pharm..

[35] Broom J., Broom A., Kirby E., Gibson A.F., Post J.J. (2017). How Do Hospital Respiratory Clinicians Perceive Antimicrobial Stewardship (AMS)? A Qualitative Study Highlighting Barriers to AMS in Respiratory Medicine. J. Hosp. Infect..

[36] Charani E., Smith I., Skodvin B., Perozziello A., Lucet J.-C., Lescure F.-X., Birgand G., Poda A., Ahmad R., Singh S. (2019). Investigating the Cultural and Contextual Determinants of Antimicrobial Stewardship Programmes across Low-, Middle-and High-Income Countries—A Qualitative Study. PLoS ONE.

[37] Currie K., Laidlaw R., Ness V., Gozdzielewska L., Malcom W., Sneddon J., Seaton R.A., Flowers P. (2020). Mechanisms Affecting the Implementation of a National Antimicrobial Stewardship Programme; Multi-Professional Perspectives Explained Using Normalisation Process Theory. Antimicrob. Resist. Infect. Control.

[38] Ierano C., Rajkhowa A., Gotterson F., Marshall C., Peel T., Ayton D., Thursky K. (2022). Opportunities for Nurse Involvement in Surgical Antimicrobial Stewardship Strategies: A Qualitative Study. Int. J. Nurs. Stud..

[39] Mula C.T., Solomon V., Muula A.S. (2019). The Examination of Nurses’ Adherence to the ‘Five Rights’ of Antibiotic Administration and Factors Influencing Their Practices: A Mixed Methods Case Study at a Tertiary Hospital, Malawi. Malawi Med. J..

[40] Nampoothiri V., Bonaconsa C., Surendran S., Mbamalu O., Nambatya W., Ahabwe Babigumira P., Ahmad R., Castro-Sanchez E., Broom A., Szymczak J. (2022). What Does Antimicrobial Stewardship Look like Where You Are? Global Narratives from Participants in a Massive Open Online Course. JAC Antimicrob. Resist..

[41] Ren-Zhang L., Chee-Lan L., Hui-Yin Y. (2020). The Awareness and Perception on Antimicrobial Stewardship among Healthcare Professionals in a Tertiary Teaching Hospital Malaysia. Arch. Pharm. Pract..

[42] van Gulik N., Hutchinson A., Considine J., Driscoll A., Malathum K., Botti M. (2021). Perceived Roles and Barriers to Nurses’ Engagement in Antimicrobial Stewardship: A Thai Qualitative Case Study. Infect. Dis. Health.

[43] Wilcock M., Powell N., Underwood F. (2019). Antimicrobial Stewardship and the Hospital Nurse and Midwife: How Do They Perceive Their Role?. Eur. J. Hosp. Pharm..

[44] Goulopoulos A., Rofe O., Kong D., Maclean A., O’Reilly M. (2019). Attitudes and Beliefs of Australian Emergency Department Clinicians on Antimicrobial Stewardship in the Emergency Department: A Qualitative Study. Emerg. Med. Australas..

[45] Monsees E., Goldman J., Popejoy L. (2017). Staff Nurses as Antimicrobial Stewards: An Integrative Literature Review. Am. J. Infect. Control.

[46] Monsees E.A., Tamma P.D., Cosgrove S.E., Miller M.A., Fabre V. (2019). Integrating Bedside Nurses into Antibiotic Stewardship: A Practical Approach. Infect. Control Hosp. Epidemiol..

[47] Fitzpatrick E.R., Pogorzelska-Maziarz M., Manning M., Gleason V.M. (2021). The Effect of an Educational Program on Nursing Knowledge and Empowerment in Antimicrobial Stewardship in a Surgical Intensive Care Unit. Dimens. Crit. Care Nurs..

[48] Greendyke W.G., Carter E.J., Salsgiver E., Bernstein D., Simon M.S., Saiman L., Calfee D.P., Furuya E.Y. (2018). Exploring the Role of the Bedside Nurse in Antimicrobial Stewardship: Survey Results From Five Acute-Care Hospitals. Infect. Control Hosp. Epidemiol..

[49] Gotterson F., Buising K., Manias E. (2021). Nurse Role and Contribution to Antimicrobial Stewardship: An Integrative Review. Int. J. Nurs. Stud..

[50] van Huizen P., Kuhn L., Russo P.L., Connell C.J. (2021). The Nurses’ Role in Antimicrobial Stewardship: A Scoping Review. Int. J. Nurs. Stud..

[51] Chater A.M., Family H., Abraao L.M., Burnett E., Castro-Sanchez E., Du Toit B., Gallagher R., Gotterson F., Manias E., McEwen J. (2022). Influences on Nurses’ Engagement in Antimicrobial Stewardship Behaviours: A Multi-Country Survey Using the Theoretical Domains Framework. J. Hosp. Infect..

[52] Mittal N., Deswal H., Mittal R., Sharma S., Kaushik P. (2023). An Educational Program on Antimicrobial Resistance and Stewardship for Staff Nurses in a Public Tertiary Care Hospital in India. Infect. Dis. Health.

[53] Gillespie E., Rodrigues A., Wright L., Williams N., Stuart R.L. (2013). Improving Antibiotic Stewardship by Involving Nurses. Am. J. Infect. Control.

[54] Castro-Sánchez E., Gilchrist M., Ahmad R., Courtenay M., Bosanquet J., Holmes A.H. (2019). Nurse Roles in Antimicrobial Stewardship: Lessons from Public Sectors Models of Acute Care Service Delivery in the United Kingdom. Antimicrob. Resist. Infect. Control.

[55] Wentzel J., van Drie-Pierik R., Nijdam L., Geesing J., Sanderman R., van Gemert-Pijnen J.E.W.C. (2016). Antibiotic Information Application Offers Nurses Quick Support. Am. J. Infect. Control.

[56] International Council of Nurses Antimicrobial Resistance Position Statement. https://www.icn.ch/sites/default/files/inline-files/PS_A_Antimicrobial_resistance_0.pdf.

[57] Filipe S., Martins T., Santos-Costa P., Paiva-Santos F., Castilho A., Bastos C. (2024). Effectiveness of A Nurse-Led Multimodal Intervention in Preventing Blood Culture Contamination: A Before-and-After Study. Healthcare.

[58] Ajzen I. (1991). The Theory of Planned Behavior. Organ. Behav. Hum. Decis. Process..

[59] Raybardhan S., Kan T., Chung B., Ferreira D., Bitton M., Shin P., Das P. (2020). Nurse Prompting for Prescriber-Led Review of Antimicrobial Use in the Critical Care Unit. Am. J. Crit. Care.

[60] Ha D.R., Forte M.B., Olans R.D., OYong K., Olans R.N., Gluckstein D.P., Kullar R., Desai M., Catipon N., Ancheta V. (2019). A Multidisciplinary Approach to Incorporate Bedside Nurses into Antimicrobial Stewardship and Infection Prevention. Jt. Comm. J. Qual. Patient Saf..

[61] Pombo M.H.R., Gandra S., Thompson D., Lamkang A.S., Pulcini C., Laxminarayan R. (2018). Global Core Standards for Hospital Antimicrobial Stewardship Programs: International Perspectives and Future Directions. https://onehealthtrust.org/wp-content/uploads/2018/12/Global-Core-Standards-for-Hospital-Antimicrobial-Stewardship-Programs.pdf.

