# Supplementary material for: Nurses’ Engagement in Antimicrobial Stewardship Programmes: A Mapping Review of Influencing Factors Based on Irvine’s Theory
Source: Nurs Rep. 2025 Jun 12;15(6):216. doi: 10.3390/nursrep15060216 (PMC12196033; doi:10.3390/nursrep15060216)
Supplement: Supplementary file 1 [file nursrep-15-00216-s001.zip › nursrep-3672225-supplementary-updated/Table S1 - Data Extraction Instrument.pdf]

nursing reports

Review

Nurses' Engagement in Antimicrobial Stewardship Programmes: A Mapping Review of Influencing Factors Based on Irvine's Theory

Susana Filipe [1,2,*], Paulo Santos-Costa [1], Celeste Bastos [3] and Amélia Castilho [1]

[1] Health Sciences Research Unit: Nursing (UICISA: E), Nursing School of Coimbra (ESEnfC), Coimbra, Portugal
[2] Local Health Unit of Baixo Mondego, Figueira da Foz, Portugal
[3] CINTESIS@RISE, Nursing School of Porto (ESEP), Porto, Portugal
* Correspondence: susanafilipe@ulsbm.min-saude.pt

Table S1. Data Extraction Tool Developed by The Researcher.

Data Extraction Tool									
Title	Nurses' Engagement in Antimicrobial Stewardship Programmes: A Mapping Review of Influencing Factors Based on Irvine's Theory								
Review question	What barriers and facilitators are reported to influence nurses' active role in AMS programmes, and what nursing-sensitive outcomes are associated with their engagement in these programmes?								
Eligibility criteria	Participants	Studies that include registered nurses practicing in healthcare. Nursing students, prescribing nurses, dental nurses, aid nurses and veterinary nurses will be excluded.							
	Concept	Studies identifying barriers and facilitators to nurses' engagement in antimicrobial stewardship programmes.							
	Context	All studies undertaken on public or private healthcare sector, or community healthcare, from any country worldwide.							
**Data Extraction Details**									
Title	Author(s)	Year of publication	Country of origin	Study design	Objective(s)	Type of nurses participation	Barriers described	Facilitators described	Nursing sensitive outcomes described
