# Supplementary material for: Nurses’ Engagement in Antimicrobial Stewardship Programmes: A Mapping Review of Influencing Factors Based on Irvine’s Theory
Source: Nurs Rep. 2025 Jun 12;15(6):216. doi: 10.3390/nursrep15060216 (PMC12196033; doi:10.3390/nursrep15060216)
Supplement: Supplementary file 1 [file nursrep-15-00216-s001.zip › nursrep-3672225-supplementary-updated/Tabel S2 - Data Extraction and Included Studies Synthesis.pdf]

nursing reports

MDPI

Review

Nurses' Engagement in Antimicrobial Stewardship Programmes: A Mapping Review of Influencing Factors Based on Irvine's Theory

Susana Filipe [1,2,*], Paulo Santos-Costa [1], Celeste Bastos [3] and Amélia Castilho [1]

1 Health Sciences Research Unit: Nursing (UICISA: E), Nursing School of Coimbra (ESEnfC), Coimbra, Portugal
2 Local Health Unit of Baixo Mondego, Figueira da Foz, Portugal
3 CINTESIS@RISE, Nursing School of Porto (ESEP), Porto, Portugal
* Correspondence: susanafilipe@ulsbm.min-saude.pt

Table S2: Data Extraction and Included Studies Synthesis

Author, country, year of publication, title	Study design	Objective(s)	Type of nurses' engagement	Barriers	Facilitators	Nursing Sensitive Outcomes
Lim, C.L. et al., Australia, 2014, Antimicrobial stewardship in residential aged care facilities: need and readiness assessment [32]	Descriptive, qualitative study with semi-structured individual and focus-group interviews	Explore the attitudes and perceptions of key healthcare providers towards antimicrobial stewardship (AMS) interventions in Australian Residential Aged Care Facilities (RACF)	Participants (among other healthcare professionals – nurses, physicians, pharmacists)	- Low staff awareness and inadequacy of existing infection control efforts in preventing MDR organisms transmission;<br>- Low awareness of the AMS concept;<br>- Lack of knowledge regarding antibiotic use;<br>- High workload;<br>- GPs acceptance of any nurses' participation in AMS interventions;<br>- Lack of awareness about potential antibiotic misuse.	- Ongoing education<br>- RACF-based guidance and support on the matter of infection management.<br><br>Nuses concerns:<br>- Frequent empiric antibiotic prescribing without microbiological investigations to confirm causative organisms.	- Not documented

Table S2: Data Extraction and Included Studies Synthesis (cont.)

Author, country, year of publication, title	Study design	Objective(s)	Type of nurses' engagement	Barriers	Facilitators	Nursing Sensitive Outcomes
Padigos, J. et al., Australia, 2023, Nursing experiences in antimicrobial optimization in the intensive care unit: a convergent analysis of a national survey [27]	Descriptive, mixed-methods study with a web-based survey	Explore nurses' perceptions and experiences of antimicrobial optimization or stewardship in ICUs in Australia	Participants	- Gaps in education;<br>- Non-inclusive antimicrobial discussions;<br>- Workload burden;<br>- Moral distress – fear of pushback or unwelcomed reactions from doctors;<br>- Lack of support from the management.	- Round discussions (mostly seen as an opportunity for nurses to engage);<br>- Educate prescribers in the contribution nurses can make to AMS tasks.	- Not documented
Ierano, C. et a., Australia, 2022, Opportunities for nurse involvement in surgical antimicrobial stewardship strategies: a qualitative study [37]	Exploratory, multi-site, collective qualitative case study (focus-group interviews)	Highlight health professionals' perspectives of Australian theatre nurses on surgical antimicrobial stewardship and relevant opportunities	Participants (among other healthcare professionals – surgeons, anesthetists, pharmacists, theatre nurses)	- Lack of role definition;<br>- Professional hierarquical structures;<br>- Lack of education and confidence in their knowledge about antimicrobials;<br>- Lack of resources and support to contribute to antimicrobial stewardship;<br>- Nurses feel unsuitable to be involved in antimicrobial decision-making;<br>- Nurses recognize their interventions as routine, not as contribution to antimicrobial stewardship;<br>- Motivation.	- Nurses do already contribute to the quality and safety of antimicrobial use;<br>- Targeted education and training;<br>- Integration of guidelines or algorithms;<br>- Multidisciplinary approach to nurses' engagement.	- Antimicrobial prophylaxis improvement, however, nurses did not perceive their involvement in this specific activity as a current priority
Hendy, A. et al, Egypt, 2023, Effect of educational interventions on nurses' perception and practice of antimicrobial stewardship programs [22]	Quasi-experimental design without control group	Evaluate the effect of the training programs on nurses' perceptions and practice of antimicrobial stewardship to embed in practice.<br>Assess challenges related to embedding practice of antimicrobial stewardship and overcome barriers from the nurses' view.	Nurse-led educational intervention	- Time constraints;<br>- Resistance to change in nurses' practice and attitude;<br>- Lack of nurses knowledge;<br>- Rapid staff rotation and turnover;<br>- Lack of education and training;<br>- Defective communication between medical staff.	- Educational interventions;<br>- Support from managers and physicians;<br>- In-service training;<br>- Continuing education.	Highlights:<br>- Improvement in knowledge about adverse effects of antimicrobial stewardship;<br>- Improvement in nurses' practice related to antibiotic preparation;<br>However, no nursing sensitive outcomes are documented.

Table S2: Data Extraction and Included Studies Synthesis (cont.)

Author, country, year of publication, title	Study design	Objective(s)	Type of nurses' engagement	Barriers	Facilitators	Nursing sensitive outcomes

Carter, E. et al., USA, 2018, Exploring the nurses' role in antibiotic stewardship: a multisite qualitative study of nurses and infection preventionists [20]	Descriptive qualitative study, with focus group and semi-structured interviews	Explore the attitudes of nurses and infection preventionists towards five of the nurse-driven antibiotic stewardship activities recommended by the ANA/CDC working group	Participants (nurses and infection preventionists)	- Nurses' limited role in antibiotic prescribing;<br>- Nurses' belief that their intervention isn't needed hence antibiotic orders are vetted by multiple personnel;<br>- Lack of knowledge (urine culture);<br>- Prescriber push back (urine culture);<br>- Lack of ongoing formal education;<br>- Lack of accountability regarding proper technique (collecting cultures);<br>- Lack of awareness of inappropriate culturing techniques impact;<br>- Belief that nurses should not be responsible for leading antibiotic time-out discussions;<br>- Lack of emphasis on the link between nursing practices and patient outcomes.	- Optimization of antibiotic as an extension of nurses' role as patient advocate;<br>- Constant presence at the patient's bedside;<br>- Education emphasizing the "why" behind proper culturing technique and the implications for patient outcomes;<br>- Educational tools.	Highlights opportunities such as:<br>- Questioning the necessity of urine culture;<br>- Ensuring proper culturing techniques;<br>- Encouraging prompt transition from intravenous to oral antibiotics;<br>However, although not documented, suggests the following outcomes:<br>- Hospitalization length of stay;<br>- Good practice in culturing technique (audits).
Wong, L. et al., Singapore, 2020, Empowerment of nurses in antibiotic stewardship: a social ecological qualitative analysis [30]	Exploratory qualitative study with focus-group discussions	Understand the facilitators and barriers that impact nurses' involvement and empowerment in antibiotic stewardship	Participants	- Lack of knowledge;<br>- Lack of patient's trust in nurses advice;<br>- Power differential with physicians;<br>- Lack of knowledge and awareness on antibiotic use and resistance among the population -> misconceptions on antibiotic treatment and poor adherence to antibiotic regimens	- Support from senior nurses;<br>- Nurse-led ward rounds;<br>- Continuing education;<br>- Assist/link in communication between physicians, patients and their caregivers;<br>- Patients and caregivers literacy;<br>- Guidelines;<br>- Awareness and empowerment in nurses' role in antibiotic administration as part of antibiotic stewardship.	- Not documented

Table S2: Data Extraction and Included Studies Synthesis (cont.)

Author, country, year of publication, title	Study design	Objective(s)	Type of nurses' engagement	Barriers	Facilitators	Nursing sensitive outcomes

Mula, C., Human, N. & Middleton, L., Malawi, 2019, An exploration of workarounds and their perceived impact on antibiotic stewardship in the adult medical wards on a referral hospital in Malawi: a qualitative study [38]	Qualitative, mixed-methods case study with focus group discussions and participant observation	Examine workarounds behaviour that nurses and doctors employ to address the challenges encountered during their antibiotic stewardship efforts.<br>Examine the impact of such behaviors.	Participants	- Staff shortage and staff constraints;<br>- Patient's level of understanding;<br>- Insufficient antibiotics;<br>- Workarounds (conceal the magnitude of nursing and antibiotic supply shortage);<br>- Unwritten communication;<br>- Lack of awareness on the importance of adhering to time intervals to minimize antibiotic resistance;<br>- Low priority activity for nurses.		- Not documented
Mustafa, Z., Paquistan, 2022, Nurses' perceptions involvement, confidence and perceived barriers towards antimicrobial stewardship program in Pakistan: findings from a multi-center, cross-sectional study [26]	Descriptive cross sectional study, with a web-based survey	Assess nurses' perception, involvement, confidence and barriers towards hospital-based antimicrobial stewardship programmes	Participants	- Time constraints (limited time);<br>- Ineffective communication with physicians;<br>- Lack of acceptance by the physicians on nurses' advice on possible antimicrobial therapy;<br>- Lack of training;<br>- Non inclusive antimicrobial discussions.		- Not documented
Davey, K. & Aveyard, UK, 2022, Nurses' perceptions of their role in antimicrobial stewardship within the hospital environment. An integrative literature review [9]	Integrative literature review	Explore the role of the nurse in AMS in order to inform education, practice and future research	(not applicable)	- Scope of practice (nurses' role);<br>- Distinction between the physicians' role in prescribing and the nurses' role in administering;<br>- Prescriber pushback (lack of confidence/ hesitancy to speak up due to fear of not being taken seriously, desire to belong to the ward team by not creating conflict);<br>- Lack of knowledge;<br>- Exclusion from ward rounds.	- Working in partnership with other health professionals;<br>- Effective communication strategies;<br>- Protocols, guidelines;<br>- Motivation (nurses feel is their duty to contribute to AMS);<br>- Education.	Highlights opportunities such as:<br>- Questioning the necessity of urine culture;<br>- Ensuring proper culturing techniques;<br>- Encouraging prompt transition from intravenous to oral antibiotics;<br>- Antibiotics time-out;<br>- Engagement in patient's and public education.<br>However, no nursing-sensitive outcomes documented.

Table S2: Data Extraction and Included Studies Synthesis (cont.)

**Author, country, year of publication, title**	**Study design**	**Objective(s)**	**Type of nurses' engagement**	**Barriers**	**Facilitators**	**Nursing sensitive outcomes**

Gotterson, F. et al., Australia, 2021, Nurse role and contribution to antimicrobial stewardship. An integrative literature review [47]	Integrative literature review	Synthetise current information about nurses' participation in antimicrobial stewardship, to identify implications for policy development, strategies to facilitate nurse involvement in stewardship, and to inform future research	(not applicable)	- Lack of awareness of AMR and AMS;<br>- Lack of education/knowledge;<br>- Distinction between the prescriber and nurses' role;<br>- Prescriber pushback;<br>- Concerns about "overstepping" professional role boundaries (complexities of hierarchy and culture);<br>- Lack of time.	- Nurse leadership;<br>- Educational interventions;<br>- Working in partnership;<br>- Educational interventions combined with behaviour change strategies;<br>- Motivation (AMS as an extension of nurses' advocacy role);<br>- Interprofessional ward rounds; Nurses formal inclusion in AMS;<br>- Nurses' influence on prescribers (although an opportunity, if not well informed can lead to bad practice); Proximity to patients; Integrating information technologies to support AMS.	Highlights opportunities such as:<br>- Questioning the necessity of urine culture;<br>- Ensuring proper culturing techniques;<br>- Encouraging prompt transition from intravenous to oral antibiotics;<br>- Good practice in antibiotic administration;<br>- Surgical prophylaxis timing;<br>- Delabelling penicillin allergies; Patient education.<br>Although no nursing-sensitive outcomes documented, the authors suggest:<br>- Days of antimicrobial;<br>- Intravenous-line days;<br>- Adequacy of urine cultures submitted for study.
Currie, K. et al., UK, 2020, Mechanisms affecting the implementation of a national antimicrobial stewardship programme: multi-professional perspectives explained using normalisation process theory [36]	Exploratory qualitative study with in-depth interviews	Explain mechanisms affecting the implementation of a national antimicrobial stewardship programme from multi-professional perspectives	Participants (among other healthcare professionals, such physicians, nurses and pharmacists)	- Lack of knowledge (insufficient training);<br>- Limited awareness of AMS;<br>- Limited involvement in AMS;<br>- Reluctance to speak-up (fear of challenging doctors' prescription); Hierarchy;<br>- AMS not viewed as a nursing role.		- Not documented
Black, E. et al., Canada, 2019, Health care providers' perceptions of antimicrobial use and stewardship at acute care hospital in Nova Scotia [33]	Descriptive, qualitative study with focus-group and individual interviews	Learn about health care providers' perceptions of current antimicrobial use and stewardship, including barriers and facilitators to improving antimicrobial use at acute care hospitals in Nova Scotia	Participants	- Prescribers resistance to nurses' recommendations;<br>- Lack of continuity of care at the organizational/system level;<br>- Gaps in knowledge		- Not documented

Table S2: Data Extraction and Included Studies Synthesis (cont.)

Author, country, year of publication, title	Study design	Objective(s)	Type of nurses' engagement	Barriers	Facilitators	Nursing Sensitive Outcomes

Broom, J. et al., Australia, 2017, How do hospital respiratory clinicians perceive antimicrobial stewardship (AMS)? A qualitative study highlighting barriers to AMS in respiratory medicine [34]	Qualitative in-depth interviews	Explore the experiences of hospital doctors and nurses regarding antibiotic use, with a focus on respiratory clinicians' perceptions of AMS interventions in a hospital setting	Participants (among other healthcare professionals – physicians, nurses)	- Limited knowledge of AMS activities and educations;<br>- Lack of perceived invitation to be engaged in AMS;<br>- Lack of awareness of the term 'antimicrobial stewardship';<br>- Reluctance to question doctor's choice of antibiotic (reluctance to cross lines);<br>- Limited engagement in AMS.	- Significant interdisciplinary and interprofessional dynamics;<br>- 'Nurses as the patient advocate' position could be utilized in interventions to optimize antibiotic use;<br>- Inclusion of appropriateness of antibiotics in nursing;<br>- Safety awareness.	Highlights opportunities such as:<br>- Encouraging prompt transition from intravenous to oral antibiotics;<br>However, no nursing-sensitive outcomes documented.
Fisher, C., Canada, 2018, A theory-informed assessment of the barriers and facilitators to nurse-driven antimicrobial stewardship [11]	Prospective. Qualitative, descriptive study, using semi-structured one-to-one interviews	Identify barriers that may be modified and facilitators that may be enhanced related to nurse promotion of iv to oral antimicrobial step-down	Participants	- Lack of knowledge;<br>- No prompts reminding nurses to assess iv to oral step-down;<br>- No standardized procedures to assess patients' elegibility;<br>- Lack of prescriber cooperation; Lack of prescriber accessibility;<br>- Lack of self-confidence;<br>- Iv to oral switch perceived as a physicians' sole role;<br>- Concerns about adverse consequences from iv to oral switch;<br>- Low priority activity for nurses.	- Capability of assessing patients iv to oral step-down;<br>- Capable of communicating results of step-down assessment to the team;<br>- Wards rounds (capable of actively participate in team activities and discuss iv to oral step-down);<br>- Familiarity with patients;<br>- Motivation (confidence in becoming leaders in promoting iv to oral step-down in ability to promote iv to oral);<br>- Resources for nurse education and training.	Highlights opportunities such as:<br>- Encouraging prompt transition from intravenous to oral antibiotics;<br>However, no nursing-sensitive outcomes documented.
Monsees, E. et al., USA, 2018, Integrating staff nurses in antibiotic stewardship: opportunities and barriers [23]	Single-centred cross-sectional survey	Identify staff nurses' perceptions and performance confidence of their stewardship role and barriers to nursing stewardship engagement	Participants	- Lack of involvement in AMS;<br>- Lack of knowledge/education;<br>- Not following procedures consistently;<br>- Poor collaboration;<br>- Poor communication;<br>- Disrespect between disciplines (culture);<br>- Variability in accepted practices or process organization;<br>- Nurses' role not well defined;<br>- Unit-based medication perceived as constraining nurses' role and responsibility double-checking;<br>- Clinical practice and hospital culture.	- Perception of being already involved/ functioning as stewards;<br>- Familiarity to the term AMS;<br>- Participation in ward rounds;<br>- Formal role to play in AMS;<br>- Enhanced education for nurses – basic microbiology, principles of antibiotic use and AMS	- Not documented

Table S2: Data Extraction and Included Studies Synthesis (cont.)

Author, country, year of publication, title	Study design	Objective(s)	Type of nurses' engagement	Barriers	Facilitators	Nursing Sensitive Outcomes

Monsees, E. et al., USA, 2019, Integrating bedside nurses into antibiotic stewardship: a practical approach [45]	Literature review	Provide a framework identifying selected practices where nurses can improve antibiotic prescribing	(not applicable)	- Unit culture:<br>o Nurses' not being included in ward rounds;<br>o Nurses' not having their input recognized or actively sought;<br>o Power differentials between disciplines;<br>- Medical hierarchy.	- Behavioural interventions;<br>- Clinical algorithms to assist nurses;<br>- Education;<br>- Strategies to enhance conversation between teams;<br>- Using an improvement model (and measure outcomes);<br>- Strengthening communication between bedside nurses and prescribers;<br>- Patient safety as an essential component of nurses work;<br>- Proximity to patients and their families.	- Not documented
Hamdy, R. et al., USA, 2019, Pediatric nurses' perceptions of their role in antimicrobial stewardship: a focus group study [21]	Qualitative, focus-group	Explore pediatric nurses' perceptions of their role in antimicrobial stewardship	Participants	- Low awareness of the term AMS;<br>- Lack of integration in AMS;<br>- Clear line between nursing activities regarding ATB prescription (physicians) and ATB administration;<br>- Deference to the physicians;<br>- Overlapping roles;<br>- Inconsistent inclusion on ward rounds;<br>- Inconsistent relationship with physicians;<br>- Consulting services may not loop in the nurse;<br>- Inconsistent and non-adherent prescribing practices;<br>- Lack of formal training.	- Perception of nurses' central role in AMS;<br>- Nurses as patient advocates (at risk of sepsis);<br>- Feel at ease to suggest alternatives or escalate concerns if needed (culture);<br>- Nurses' experience and knowledge of institutional protocols (guidelines);<br>- Communication with the team – communicating updates on patient status and medication adverse events, communicating with other nurses);<br>- Education.	Highlights opportunities such as:<br>- Reducing medication errors;<br>- Educating patients and family/caregivers;<br>However, no nursing-sensitive outcomes documented.
Rout, J. & Brysiewick, P. , South Africa, 2020, Perceived barriers to the development of the antimicrobial stewardship role of the nurse in intensive care: views of healthcare professionals [12]	Qualitative descriptive study, with individual interviews	Explore the views of healthcare professionals regarding barriers to the antimicrobial stewardship role of the nurse in intensive care in a private hospital in Kwa-Zulu-Natal, South Africa	Participants (among other healthcare professionals, such as nurses, physicians and pharmacists)	- Lack of collaboration:<br>o Not participating in the AMS programme;<br>o No feedback on AMR;<br>o Not part of decision-making;<br>- Inadequate knowledge:<br>o Not understanding IPC;<br>o Missing the link between laboratory results and start of treatment;<br>- Inexperienced nurses:<br>o Inadequate nursing staff.	- Suggested strategies:<br>o Development of nurses role in AMS;<br>o Need for diplomacy;<br>o Include bedside nurses in AMS interventions;<br>o Allow opportunities for learning;<br>o Identify ICU trained nurses working in this ICU in order to facilitate communication between members of the AMS team.	- Not documented

Table S2: Data Extraction and Included Studies Synthesis (cont.)

**Author, country, year of publication, title**	**Study design**	**Objective(s)**	**Type of nurses' engagement**	**Barriers**	**Facilitators**	**Nursing Sensitive Outcomes**

Van Gulick, N. et al., Thailand, 2021, Perceived roles and barriers to nurses' engagement in antimicrobial stewardship: a Thai qualitative case study [41]	Qualitative, descriptive study, with individual interviews	Explore how organizational multidisciplinary leaders and clinical nurses' roles in AMS in a single organizational site case study based in Thailand within the current governance, educational and practice context and the barriers to nurses' engagement in AMS	Participants	- Poor articulation of nurses' roles in policies;<br>- Practice cultures (traditional professional hierarchies);<br>- Inconsistent nurses' engagement in AMS related activities;<br>- Information technology systems not conductive;<br>- Inadequate knowledge of the principles of ATB use;<br>- Shared perception that since nurses do not prescribe ATB, AMS is not their primary responsibility.	- Nurses with specialty knowledge are recognized participants and leaders in AMS;<br>- Working collaboratively with pharmacists.	Highlights opportunities such as:<br>- Identification and documentation of medication allergies;<br>- Monitoring signs of infection;<br>- Surgical prophylaxis timing;<br>- Encouraging prompt transition from intravenous to oral antibiotics;<br>- Antibiotic time-out;<br>Although no nursing-sensitive outcomes documented.
Groumoutis, J. et al., Canada, 2023, Identifying opportunities for antimicrobial stewardship in a tertiary intensive care unit: a qualitative study [31]	Qualitative semi-structured one to one interviews	Understand ICU nursing and physician priorities and preferences around AMS and possible AMS interventions for implementation in the ICU	Participants	- Lack of knowledge;<br>- Lack of awareness of the existing AMS programmes and nurses' role;<br>- Nurses did not generally believe stewardship to be a nursing role;<br>- High workload (delay in collecting specimens).	- Thorough communication of the antimicrobial treatment plan between healthcare professionals;<br>- Designated AMS nurse champion;<br>- Algorithms and pathways;<br>- Communication improvement;<br>- Participation on rounds.	- Not documented
Charani, E. et al., UK, 2019, Investigating the cultural and contextual determinants of antimicrobial stewardship programmes across low-, middle- and high- income countries – a qualitative study [35]	Qualitative, face-to-face interviews using Grounded Theory approach	Map the key contextual, including cultural, drivers of the development and implementation of ASP across different resource settings.	Participants	- Professional boundaries and hierarchies;<br>- Lack of engagement with the wider healthcare force.		- Not documented
Zhao, W. et al., China, 2023, Bedside nurses' antimicrobial stewardship practice scope and competencies in acute hospital settings: a scoping review [14]	Scoping review	Identifying and map bedside nurses' practice scope and competencies regarding antimicrobial stewardship in acute hospital settings, and develop a competency framework for them.	(not applicable)	- Lack of knowledge about AMS;<br>- Lack of confidence;<br>- Unclear understanding of nurses' role and practices.	- Specialized knowledge;<br>- Patient advocates;<br>- Strategies to enhance dialogue between teams (SBAR tool);<br>- Nurses' capability of influencing prescribers' decisions;<br>- Effective communication and team work.	Highlights opportunities such as:<br>- Identification and documentation of medication allergies;<br>- Safe antibiotic administration process;<br>- Question the need for urine culture;<br>- Proper culture collection technique;<br>- Antibiotic time-out;<br>Nursing-sensitive outcomes identified:<br>- Device-associated infection rate, such as central-line associated blood stream infection and catheter-associated urinary tract infection.

Table S2: Data Extraction and Included Studies Synthesis (cont.)

**Author, country, year of publication, title**	**Study design**	**Objective(s)**	**Type of nurses' engagement**	**Barriers**	**Facilitators**	**Nursing Sensitive Outcomes**

Nampoothiri, V. et al., UK, 2021, What does antimicrobial stewardship look like where you are? Global narrative from participants in a massive open online course [39]	Qualitative, content analysis	Describe how the use of a massive open online course (MOOC) platform provided an opportunity to gather diverse narratives on AMS from around the world	Participants	- Nurses' role still ill defined;<br>- Unavailability of AMS-specific policies and protocols;<br>- Lack of education/training in AMS;<br>- Inadequacy of information systems;<br>- Limited engagement in AMS teams.	- Nurses' active role educating patients;<br>- Delivering AMS through a multidisciplinary team (viewed to be desirable);<br>- Local championship and leadership.	- Not documented
Fitzpatrick, Eleanor R., USA, 2021, The Effect of an Educational Program on Nursing Knowledge and Empowerment in Antimicrobial Stewardship in a Surgical Intensive Care Unit [47]	Quasi-experimental pre-post study	Evaluate the impact of an educational intervention on AMS knowledge and sense of empowerment among bedside registered nurses in a surgical intensive care unit in an academic medical center	Participants	- Lack of knowledge (ATB);<br>- Sense of not being heard;<br>- Other team members may not be in support of nursing involvement in AMS	- Training and education;<br>- Leadership support;<br>- Institutional culture.	- Not documented
Ren-Zhang, L. et al., Malaysia, 2020, The awareness and perception on Antimicrobial Stewardship among healthcare professionals in a tertiary teaching hospital Malaysia [40]	Cross-sectional study	Assessing the awareness and perception of AMS among doctors and nurses across various disciplines in the UKMMC	Participants	- Unfamiliar with the term AMS;<br>- Knowledge gap on AMR and AMS;<br>- Under-recognition;<br>- Hierarchy/culture;<br>- Not perceiving themselves as playing a role in implementing AMS;<br>- Nurses were neutral about the potential outcomes of AMS;<br>- Low awareness on AMS.	- Training and education for nurses;<br>- Support to take up leadership;<br>- Promotional activities for AMS strategies.	- Not documented
Van Huizen, P. et al., Australia, 2020, The nurses' role in antimicrobial stewardship: a scoping review [48]	Scoping review	Investigate the nurses' role in antimicrobial stewardship and examine best practice for preparing, administering and disposing of intravenous antibiotics	(not applicable)	- Adding new responsibilities may be considered as competing demands on nurses' time and resources;<br>- Low level of understanding the term antimicrobial stewardship.	- Education;<br>- AMS already is part of the nurses' role;<br>- Improved skills in communicating with prescribers and pharmacists;<br>- Awareness campaigns;<br>- Local, national and international support.	- Medication errors, however, limited research describing best practice when preparing, administering and disposing of intravenous antibiotics.

Table S2: Data Extraction and Included Studies Synthesis (cont.)

Author, country, year of publication, title	Study design	Objective(s)	Type of nurses' engagement	Barriers	Facilitators	Nursing Sensitive Outcomes

Monsees, E., et al., USA, 2017, Staff nurses as antimicrobial stewards: an integrative literature review [44]	Integrative literature review	Examine the extent literature on the role of staff nurses in antibiotic stewardship to develop strategies to enhance nursing participation in ASPs.	(not applicable)	- Lack of resources to participate meaningfully in ASP;<br>- Lack of recognition on nurses' contribution;<br>- Knowledge gaps.	- Consistent presence across the care continuum;<br>- Focused education on ASP.	- Not documented
Wilcock et al. UK, 2019, Antimicrobial stewardship and the hospital nurse and midwife: how do they perceive their role [42]	On-line survey	Determine the views of nurses and midwives in an acute hospital regarding a potential role in an antimicrobial stewardship programme	Participants	- Time constraints/workload;<br>- Lack of knowledge/keeping knowledge up to date;<br>- Lack of adequate staff education/training;<br>- Lack of confidence in challenging medical staff;<br>- Changing practice/habits/attitudes;<br>- Patient/family expectations;<br>- Not sure this is relevant to nurses' role.	- Support needed to take forward an AS role on the ward;<br>- Protected time for teaching/learning;<br>- Expert contact/mentor.	- Not documented
Monsees, E. et al., USA, 2020, Nurses' as antimicrobial stewards: recognition, confidence and organizational factors across mime hospitals [25]	Descriptive, quantitative, survey	Identify nurses' understanding of AS clinical practices, associated confidence with processes and the latent influence of organizational culture on nurses AS	Participants	- Lack of knowledge (gap);<br>- Professional boundaries;<br>- Competing demands;<br>- Unrecognized contribution;<br>- Focus on medication administration;<br>- Disregard for contribution.	- Formal education;<br>- Empowerment techniques to enhance communication;<br>- Soliciting nurses' input;<br>- Algorithms.	- Not documented
Hansen, M., Norway, 2023, Attitudes and self-efficacy towards infection prevention and control and antibiotic stewardship among nurses: a mixed-methods study [29]	Descriptive, convergent, mixed-methods study with quantitative and qualitative approach	Gain a more comprehensive understanding of nurses' IPC and AS attitudes and self-efficacy when caring for patients with MDRB in a hospital setting	Participants	- Lack of knowledge on AMS;<br>- Unclear role;<br>- Reluctance to speak-up on antibiotic treatment or on iv to oral switch;<br>- Lack of training on AMS.	- High self-efficacy perspective;<br>- Active participation in clinical observations, microbiological testing and administering antibiotics;<br>- Independent nursing interventions perceived as valuable within the multidisciplinary AMS team.	- Not documented
Goulopoulos, A., Australia, 2019, Attitudes and beliefs of Australian emergency department clinicians on antimicrobial stewardship in the emergency department: a qualitative study [43]	Descriptive qualitative study with semi-structured one-to-one interviews	Explore the attitudes and beliefs of Australian emergency department (ED) clinicians towards antimicrobial stewardship in ED	Participants	- Lack of education and knowledge of antimicrobials;<br>- Poor communication regarding antimicrobial prescription impacts timely administration;<br>- Staff shortage;<br>- Time pressures (transfer patients to wards resulted in administration delays);<br>- Physicians' pushback;<br>- Lack of easy access guidelines.	- Improved interprofessional communication (of newly prescribed antimicrobials would improve timely administration);<br>- Robust, readily, available, easily accessible, up-to-date, indication-specifics and non-conflicting guidelines;<br>- Continuing education.	- Not documented

Table S2: Data Extraction and Included Studies Synthesis (cont.)

Author, country, year of publication, title	Study design	Objective(s)	Type of nurses' engagement	Barriers	Facilitators	Nursing Sensitive Outcomes
Mostaghim, M. et al., Australia, 2017, Nurses	Quantitative, descriptive study	Explore perceptions and attitudes of nurses in regard to	Participants	- Lack of knowledge;	- Patient advocacy;	- Not documented

are underutilized in antimicrobial stewardship: results of a multisite survey in pediatric and adult hospital [25]		antimicrobial stewardship and their role as nurses.<br>Identify differences in perceptions and attitudes across pediatric and adult settings.		- Nurses' role in AMS needs clarification;<br>- Institutional/hierarquical support;<br>- Low/few participation in multidisciplinary discussions;<br>- Feeling of challenging prescribers' decision.	- Targeted education on antimicrobial use;<br>- Motivation (willingness to question inappropriate prescribing).	
