# Supplementary material for: Nurses’ Engagement in Antimicrobial Stewardship Programmes: A Mapping Review of Influencing Factors Based on Irvine’s Theory
Source: Nurs Rep. 2025 Jun 12;15(6):216. doi: 10.3390/nursrep15060216 (PMC12196033; doi:10.3390/nursrep15060216)
Supplement: Supplementary file 1 [file nursrep-15-00216-s001.zip › nursrep-3672225-supplementary-updated/Table S3 - Full-text studies that did not meet the inclusion criteria.pdf]

nursing reports

Nurses' Perceived Barriers and Facilitators to Engage in Antimicrobial Stewardship: A Scoping Review

Supplemental Digital Content

Full-text studies that did not meet the inclusion criteria

Reason 1: Did not address the concept of interest

- Schellack, N., Pretorius, R., & Messina, A. P. (2016). 'Esprit de corps': Towards collaborative integration of pharmacists and nurses into antimicrobial stewardship programmes in South Africa. *South African medical journal = Suid-Afrikaanse tydskrif vir geneeskunde*, *106*(10), 973–974. https://doi.org/10.7196/SAMJ.2016.v106i10.11468
- Mittal, N., Deswal, H., Mittal, R., Sharma, S., & Kaushik, P. (2023). An educational program on antimicrobial resistance and stewardship for staff nurses in a public tertiary care hospital in India. *Infection, disease & health*, *28*(3), 202–210. https://doi.org/10.1016/j.idh.2023.03.003
- Santillo, M., Sivyer, K., Krusche, A., Mowbray, F., Jones, N., Peto, T. E. A., Walker, A. S., Llewelyn, M. J., Yardley, L., & ARK-Hospital (2019). Intervention planning for Antibiotic Review Kit (ARK): a digital and behavioural intervention to safely review and reduce antibiotic prescriptions in acute and general medicine. *The Journal of antimicrobial chemotherapy*, *74*(11), 3362–3370. https://doi.org/10.1093/jac/dkz333
- Jeffs, L., Law, M. P., Zahradnik, M., Steinberg, M., Maione, M., Jorgoni, L., Bell, C. M., & Morris, A. M. (2018). Engaging Nurses in Optimizing Antimicrobial Use in ICUs: A Qualitative Study. *Journal of nursing care quality*, *33*(2), 173–179. https://doi.org/10.1097/NCQ.0000000000000281
- Hamidi, M., & Blatz, M. A. (2022). Antimicrobial Stewardship Programs in Preterm Infants: Encouraging Staff Nurse Engagement. *Neonatal network: NN*, *41*(6), 319–324. https://doi.org/10.1891/NN-2021-0022
- Felix AM da S, Toffolo SR. Participation of nurses in antimicrobial stewardship programs: an integrative review. Cogitare enferm. [Internet]. 2019; 24. Available at: http://dx.doi.org/10.5380/ ce.v24i0.59324
- Cotta, M.O., Robertson, M.S., Tacey, M., Marshall, C., Thursky, K. A., Liew, D. & Buising, K.L. (2014). Attitudes towards antimicrobial stewardship: results from a large private hospital in Australia. Healthcare Infection, 19, 89-94. http://dx.doi.org/10.1071/HI14008
- Chaaban, T., Ahouah, M., Lombrail, P., Morvillers, J. M., Rothan-Tondeur, M., & Carroll, K. (2019). Nursing Role for Medication Stewardship Within Long-Term Care Facilities. *Nursing science quarterly*, *32*(2), 113–115. https://doi.org/10.1177/0894318419826310
- Strahilevitz, J., Oreg, S., Nir Paz, R., & Sagiv, L. (2022). Nurses' and Physicians' Responses to a New Active Antimicrobial Stewardship Program: A Two-Phase Study of Responses and Their Underlying Perceptions and Values. *International journal of health policy and management*, *11*(12), 2982–2989. https://doi.org/10.34172/ijhpm.2022.6557
- Raybardhan, S., Kan, T., Chung, B., Ferreira, D., Bitton, M., Shin, P., & Das, P. (2020). Nurse Prompting for Prescriber-Led Review of Antimicrobial Use in the Critical Care Unit. *American journal of critical care : an official publication, American Association of Critical-Care Nurses*, *29*(1), 71–76. https://doi.org/10.4037/ajcc2020272
- Padigos, J., Reid, S., Kirby, E., & Broom, J. (2021). Knowledge, perceptions and experiences of nurses in antimicrobial optimization or stewardship in the intensive care unit. *The Journal of hospital infection*, *109*, 10–28. https://doi.org/10.1016/j.jhin.2020.12.003
- Abbas, S., Lee, K., Pakyz, A., Markley, D., Cooper, K., Vanhoozer, G., Doll, M., Bearman, G., & Stevens, M. P. (2019). Knowledge, attitudes, and practices of bedside nursing staff regarding antibiotic stewardship: A cross-sectional study. *American journal of infection control*, *47*(3), 230–233. https://doi.org/10.1016/j.ajic.2018.09.008

- Adegbite, B. R., Edoa, J. R., Schaumburg, F., Alabi, A. S., Adegnika, A. A., & Grobusch, M. P. (2022). Knowledge and perception on antimicrobial resistance and antibiotics prescribing attitude among physicians and nurses in Lambaréné region, Gabon: a call for setting-up an antimicrobial stewardship program. *Antimicrobial resistance and infection control*, *11*(1), 44. https://doi.org/10.1186/s13756-022-01079-x
- Gulleen, E. A., Lubwama, M., Komakech, A., Krantz, E. M., Liu, C., & Phipps, W. (2022). Knowledge and perceptions of antimicrobial resistance and antimicrobial stewardship among staff at a national cancer referral center in Uganda. *Antimicrobial stewardship & healthcare epidemiology : ASHE*, *2*(1), e54. https://doi.org/10.1017/ash.2022.28
- Singh, S., Mendelson, M., Surendran, S., Bonaconsa, C., Mbamalu, O., Nampoothiri, V., Boutall, A., Hampton, M., Dhar, P., Pennel, T., Tarrant, C., Leather, A., Holmes, A., Charani, E., & ASPIRES co-investigators (2021). Investigating infection management and antimicrobial stewardship in surgery: a qualitative study from India and South Africa. *Clinical microbiology and infection : the official publication of the European Society of Clinical Microbiology and Infectious Diseases*, *27*(10), 1455–1464. https://doi.org/10.1016/j.cmi.2020.12.013
- Ierano, C., Thursky, K., Peel, T., Rajkhowa, A., Marshall, C., & Ayton, D. (2019). Influences on surgical antimicrobial prophylaxis decision making by surgical craft groups, anaesthetists, pharmacists and nurses in public and private hospitals. *PloS one*, *14*(11), e0225011. https://doi.org/10.1371/journal.pone.0225011
- Chater, A. M., Family, H., Abraao, L. M., Burnett, E., Castro-Sanchez, E., Du Toit, B., Gallagher, R., Gotterson, F., Manias, E., McEwen, J., Moralez de Figueiredo, R., Nathan, M., Ness, V., Olans, R., Padoveze, M. C., & Courtenay, M. (2022). Influences on nurses' engagement in antimicrobial stewardship behaviours: a multi-country survey using the Theoretical Domains Framework. *The Journal of hospital infection*, *129*, 171–180. https://doi.org/10.1016/j.jhin.2022.07.010
- Rout J, Brysiewicz P. Exploring the role of the ICU nurse in the antimicrobial stewardship team at a private hospital in KwaZulu-Natal, South Africa. *South Afr J Crit Care.* 2017;33(2):46–50. https://doi.org/10.7196/SAJCC.2017.v33i2.331
- Mei-Sheng Riley, M., & Olans, R. (2021). Implementing an Antimicrobial Stewardship Program in the Intensive Care Unit by Engaging Critical Care Nurses. *Critical care nursing clinics of North America*, *33*(4), 369–380. https://doi.org/10.1016/j.cnc.2021.07.001
- Singh, S., Degeling, C., Fernandez, D., Montgomery, A., Caputi, P., & Deane, F. P. (2022). How do aged-care staff feel about antimicrobial stewardship? A systematic review of staff attitudes in long-term residential aged-care. *Antimicrobial resistance and infection control*, *11*(1), 92. https://doi.org/10.1186/s13756-022-01128-5
- Rout, J., Essack, S., & Brysiewicz, P. (2021). Guidelines for the hospital role of the clinical nurse in antimicrobial stewardship: A scoping review. *The Southern African journal of critical care : the official journal of the Critical Care Society*, *37*(2), 10.7196/SAJCC.2021.v37i2.481. https://doi.org/10.7196/SAJCC.2021.v37i2.481
- Ju, J., Han, K., Ryu, J., & Cho, H. (2022). Nurses' attitudes toward antimicrobial stewardship in South Korea. *The Journal of hospital infection*, *129*, 162–170. https://doi.org/10.1016/j.jhin.2022.07.016
- Ha, D. R., Forte, M. B., Olans, R. D., OYong, K., Olans, R. N., Gluckstein, D. P., Kullar, R., Desai, M., Catipon, N., Ancheta, V., Lira, D., Khattak, Y., Legge, J., Nguyen, K. B., Chan, S., Mourani, J., & McKinnell, J. A. (2019). A Multidisciplinary Approach to Incorporate Bedside Nurses into Antimicrobial Stewardship and Infection Prevention. *Joint Commission journal on quality and patient safety*, *45*(9), 600–605. https://doi.org/10.1016/j.jcjq.2019.03.003
- Lim, S. H., Bouchoucha, S. L., Aloweni, F., & Bte Suhari, N. (2021). Evaluating knowledge and perception of antimicrobial stewardship among nurses in an acute care hospital. *Infection, disease & health*, *26*(3), 228–232. https://doi.org/10.1016/j.idh.2021.02.002
- Edwards, R., Drumright, L., Kiernan, M., & Holmes, A. (2011). Covering more Territory to Fight Resistance: Considering Nurses' Role in Antimicrobial Stewardship. *Journal of infection prevention*, *12*(1), 6–10. https://doi.org/10.1177/1757177410389627
- Manning, M. L., Pogorzelska-Maziarz, M., Hou, C., Vyas, N., Kraemer, M., Carter, E., & Monsees, E. (2022). A novel framework to guide antibiotic stewardship nursing practice. *American journal of infection control*, *50*(1), 99–104. https://doi.org/10.1016/j.ajic.2021.08.029

- Monsees, E., Lee, B., Wirtz, A., & Goldman, J. (2020). Implementation of a nurse-driven antibiotic engagement tool in 3 hospitals. *American journal of infection control*, *48*(12), 1415–1421. https://doi.org/10.1016/j.ajic.2020.07.002
- Ackers, L., Ackers-Johnson, G., Seekles, M., Odur, J., & Opio, S. (2020). Opportunities and Challenges for Improving Anti-Microbial Stewardship in Low- and Middle-Income Countries; Lessons Learnt from the Maternal Sepsis Intervention in Western Uganda. *Antibiotics (Basel, Switzerland)*, *9*(6), 315. https://doi.org/10.3390/antibiotics9060315
- Soares AR (2018). O contributo dos enfermeiros na Antibiotic Stewardship: perceções, atitudes e conhecimentos de um grupo de enfermeiros portugueses [Internet]. 2018. Available from: http://hdl.handle.net/10362/52477
- Sutthiruk, N., Considine, J., Hutchinson, A., Driscoll, A., Malathum, K., & Botti, M. (2018). Thai clinicians' attitudes toward antimicrobial stewardship programs. *American journal of infection control*, *46*(4), 425–430. https://doi.org/10.1016/j.ajic.2017.09.022
- Sakaguchi, M., Aminaka, M., & Nishioka, M. (2023). The roles of bedside nurses in Japan in antimicrobial stewardship. *American journal of infection control*, *51*(1), 48–55. https://doi.org/10.1016/j.ajic.2022.02.026
- Bonaconsa C, Mbamalu O, Mendelson M Groote Schuur Hospital Antimicrobial Stewardship and Surgical Study Group*, et al.* Visual mapping of team dynamics and communication patterns on surgical ward rounds: an ethnographic study. *BMJ Quality & Safety* 2021;**30:**812-824. https://doi.org/10.1136/bmjqs-2020-012372
- Thurman Johnson, C., Ridge, L. J., & Hessels, A. J. (2023). Nurse Engagement in Antibiotic Stewardship Programs: A Scoping Review of the Literature. *Journal for healthcare quality : official publication of the National Association for Healthcare Quality*, *45*(2), 69–82. https://doi.org/10.1097/JHQ.0000000000000372
- Hamidi, M., & Blatz, M. A. (2023). A National Survey of Neonatal Nurses' Knowledge, Beliefs, and Practices of Antibiotic Stewardship. *Advances in neonatal care : official journal of the National Association of Neonatal Nurses*, *23*(1), E22–E28. https://doi.org/10.1097/ANC.0000000000001019
- Manning, M. L., Fitzpatrick, E., Delengowski, A. M., Hou, C. M., Vyas, N., & Pogorzelska-Maziarz, M. (2022). Advancing Antibiotic Stewardship Nursing Practice Through Standardized Education: A Pilot Study. *Journal of continuing education in nursing*, *53*(9), 417–423. https://doi.org/10.3928/00220124-20220805-08
- Hamilton, R. M., Merrill, K. C., Luthy, K. E., & Nuttall, C. (2020). Knowledge, attitudes, and perceptions of nurse practitioners about antibiotic stewardship. *Journal of the American Association of Nurse Practitioners*, *33*(11), 909–915. https://doi.org/10.1097/JXX.0000000000000467
- Castro-Sánchez, E., Gilchrist, M., Ahmad, R., Courtenay, M., Bosanquet, J., & Holmes, A. H. (2019). Nurse roles in antimicrobial stewardship: lessons from public sectors models of acute care service delivery in the United Kingdom. *Antimicrobial resistance and infection control*, *8*, 162. https://doi.org/10.1186/s13756-019-0621-4

Reason 2: Did not address the population of interest

- Baubie, K., Shaughnessy, C., Kostiuk, L., Varsha Joseph, M., Safdar, N., Singh, S. K., Siraj, D., Sethi, A., & Keating, J. (2019). Evaluating antibiotic stewardship in a tertiary care hospital in Kerala, India: a qualitative interview study. *BMJ open*, *9*(5), e026193. https://doi.org/10.1136/bmjopen-2018-026193
- Baraka, M. A., Alsultan, H., Alsalman, T., Alaithan, H., Islam, M. A., & Alasseri, A. A. (2019). Health care providers' perceptions regarding antimicrobial stewardship programs (AMS) implementation-facilitators and challenges: a cross-sectional study in the Eastern province of Saudi Arabia. *Annals of clinical microbiology and antimicrobials*, *18*(1), 26. https://doi.org/10.1186/s12941-019-0325-x
- Peel, T. N., Watson, E., Cairns, K., Lam, H. Y. A., Li, H. Z., Ravindran, G., Seneviratne, J., Daly, D., Liew, S., McGiffin, D., Myles, P., & Ayton, D. (2020). Perioperative antimicrobial decision making: Focused ethnography study in orthopedic and cardiothoracic surgeries in an Australian hospital. *Infection control and hospital epidemiology*, *41*(6), 645–652. https://doi.org/10.1017/ice.2020.48

- Hall, J., Hawkins, O., Montgomery, A., Singh, S., Mullan, J., & Degeling, C. (2022). Dismantling antibiotic infrastructures in residential aged care: The invisible work of antimicrobial stewardship (AMS). *Social science & medicine (1982)*, *305*, 115094. https://doi.org/10.1016/j.socscimed.2022.115094
- Burton, E., O'Driscoll, M., & Fleming, A. (2022). The protected antimicrobial process in a University Teaching Hospital: a qualitative interview study exploring the knowledge, attitudes, and experiences of healthcare professionals. *International journal of clinical pharmacy*, *44*(3), 630–640. https://doi.org/10.1007/s11096-022-01381-z
- Lee, M. S. L., & Stead, W. (2022). A Seat at the Table: Delivering Effective Infectious Diseases and Antimicrobial Stewardship Education to Advanced Practice Providers at an Academic Medical Center. *The Journal of continuing education in the health professions*, *42*(1), e27–e31. https://doi.org/10.1097/CEH.0000000000000383

Reason 3: Did not address both the population and concept of interest

- Jha, N., Mudvari, A., Hayat, K., & Shankar, P. R. (2023). Perceptions Regarding Antimicrobial Resistance and Stewardship Programs among Healthcare Professionals. *Journal of Nepal Health Research Council*, *20*(3), 689–696. https://doi.org/10.33314/jnhrc.v20i3.3992
- Briquet, C., Khaouch, Y., & Yombi, J. C. (2023). Perceptions, attitudes, and practices of a Belgian teaching hospital's physicians, pharmacists, and nurses regarding antibiotic use and resistance: survey towards targeted actions for Antimicrobial Stewardship. *Antimicrobial resistance and infection control*, *12*(1), 19. https://doi.org/10.1186/s13756-023-01228-w
- Ramly, E., Tong, M., Bondar, S., Ford, J. H., 2nd, Nace, D. A., & Crnich, C. J. (2020). Workflow Barriers and Strategies to Reduce Antibiotic Overuse in Nursing Homes. *Journal of the American Geriatrics Society*, *68*(10), 2222–2231. https://doi.org/10.1111/jgs.16632
